# ShlA toxin of *Serratia* induces P2Y2- and α5β1-dependent autophagy and bacterial clearance from host cells

**DOI:** 10.1016/j.jbc.2023.105119

**Published:** 2023-07-30

**Authors:** Marisel R. Tuttobene, Julieta Schachter, Cora L. Álvarez, Nicolás A. Saffioti, M. Florencia Leal Denis, Horst Kessler, Eleonora García Véscovi, Pablo J. Schwarzbaum

**Affiliations:** 1 Instituto de Biología Molecular y Celular de Rosario, Consejo Nacional de Investigaciones Científicas y Tecnológicas, Universidad Nacional de Rosario, Rosario, Argentina; 2 Facultad de Farmacia y Bioquímica, Instituto de Química y Físico-Química Biológicas “Prof. Alejandro C. Paladini”, Consejo Nacional de Investigaciones Científicas y Técnicas (CONICET), Universidad de Buenos Aires (UBA), Buenos Aires, Argentina; 3 Facultad de Farmacia y Bioquímica, Departamento de Química Biológica, Universidad de Buenos Aires (UBA), Buenos Aires, Argentina; 4 Instituto de Nanosistemas, Universidad Nacional de General San Martín, Buenos Aires, Argentina; 5 Department Chemie, Institute for Advanced Study, Technical University Munich, Garching, Germany

**Keywords:** bacterial infections, Serratia, integrins, purinergic signaling, extracellular ATP, ectonucleotidases

## Abstract

*Serratia marcescens* is an opportunistic human pathogen involved in antibiotic-resistant hospital acquired infections. Upon contact with the host epithelial cell and prior to internalization, *Serratia* induces an early autophagic response that is entirely dependent on the ShlA toxin. Once *Serratia* invades the eukaryotic cell and multiples inside an intracellular vacuole, ShlA expression also promotes an exocytic event that allows bacterial egress from the host cell without compromising its integrity. Several toxins, including ShlA, were shown to induce ATP efflux from eukaryotic cells. Here, we demonstrate that ShlA triggered a nonlytic release of ATP from Chinese hamster ovary (CHO) cells. Enzymatic removal of accumulated extracellular ATP (eATP) or pharmacological blockage of the eATP-P2Y2 purinergic receptor inhibited the ShlA-promoted autophagic response in CHO cells. Despite the intrinsic ecto-ATPase activity of CHO cells, the effective concentration and kinetic profile of eATP was consistent with the established affinity of the P2Y2 receptor and the known kinetics of autophagy induction. Moreover, eATP removal or P2Y2 receptor inhibition also suppressed the ShlA-induced exocytic expulsion of the bacteria from the host cell. Blocking α5β1 integrin highly inhibited ShlA-dependent autophagy, a result consistent with α5β1 transactivation by the P2Y2 receptor. In sum, eATP operates as the key signaling molecule that allows the eukaryotic cell to detect the challenge imposed by the contact with the ShlA toxin. Stimulation of P2Y2-dependent pathways evokes the activation of a defensive response to counteract cell damage and promotes the nonlytic clearance of the pathogen from the infected cell.

*Serratia marcescens* is an opportunistic pathogen that can lead to life-threatening disease such as meningitis, endocarditis, pneumonia, and bacteremia that may lead to sepsis (1, 2, 3). The increasing incidence of *S. marcescens* in clinical settings is due to the expression of various virulence factors, the acquisition of multiple antibiotic resistances, and the ability of the pathogen to resist disinfection procedures (4, 5, 6, 7). The World Health Organization classified *S. marcescens* among the pathogens that are a research priority to design alternative antimicrobial strategies (8). Therefore, the understanding of the molecular mechanism of action of *Serratia* virulence effectors will lead to the rational design of novel antibacterial therapies.

*S. marcescens* expresses and releases the ShlA toxin to the surface of the bacteria, where it can get in contact with host eukaryotic cells (9, 10). The *shlBA* operon encodes the Type Vb two-partner secretion system composed by the ShlB translocator and by ShlA (11, 12). As a type V secretion system-delivered effector, ShlA does not show homology to other class of cytolysins such as the repeats in toxin or the cholesterol-depending cytolysins pore-forming toxins (13). ShlA exerts a cytotoxic action on erythrocytes, fibroblasts, and epithelial cells (14, 15). In animal models of infection, strains lacking ShlA expression are strongly attenuated in their pathogenic phenotypes (16, 17, 18).

Autophagy (AP) is a key cellular quality control process in eukaryotes being engaged in normal physiology and to counter diverse forms of cellular stress. Although certain microbes are able to hijack the autophagic process to their own benefit, the autophagic response to microbial invaders includes the removal of the pathogen and its virulence effectors (19). We have previously shown that ShlA is able to trigger a reversible autophagic response before *Serratia* internalization in epithelial cells (20).

After internalization, *S. marcescens* resides and proliferates in autophagic-like vacuoles and is able to avoid lysosomal elimination (21). Later on, intravacuolar *Serratia* egresses from the host cell by provoking an ShlA-dependent nonlytic, exocytic mechanism (22), which eliminates the multiplied pathogen from the infected cell but allows bacterial extracellular dissemination.

ShlA was previously shown to induce ATP depletion from epithelial cells and fibroblasts (14), but the mechanisms mediating ATP efflux and the resulting accumulation of extracellular ATP (eATP) on the infection process were not studied. Intracellular ATP (iATP) can be released from different cell types by calcium-regulated exocytosis, membrane transporters, and channels, as well as by cell lysis (23, 24, 25). On the other hand, an important conduit-mediating ATP release is the pannexon, homoheptamer of pannexin1 (PNX1), a protein expressed in the cell membranes of many cell types (26). Depending on the cell type, stimulus, and metabolic status, different ATP conduits can be activated (27).

Most cellular responses to eATP and other nucleotides are mediated by purinergic P2 receptors, classified as P2X and P2Y (28). *In vivo*, all P2X receptors are exclusively activated by eATP and mediate the transport of Na^+^, K^+^, and Ca^2+^ across the plasma membrane. eATP can also bind P2Y receptors that activate G proteins, promoting changes in the concentrations of cytosolic calcium and/or cAMP and downstream signaling routes (28). The strength and duration of P2Y receptor responses are controlled by ectonucleotidases, which usually maintain very low eATP in the pericellular space (29).

Purinergic signaling can be used by the host to activate defense mechanisms, as well as by pathogens to subvert cytoprotective strategies of the eukaryotic cell (30, 31). On one hand, eATP, released as a danger signal by injured or stressed cells, plays an important role in the regulation of immune responses, as it triggers purinergic-dependent release of proinflammatory cytokines and chemokines and cell repair processes. On the other hand, toxins such as those that belong to the repeats in toxin-family are known to induce ATP release and the stimulation of P2 receptors that permeabilize the plasma membrane to diffusible ions, and can cause swelling-dependent cell lysis (23, 32, 33, 34).

These findings point out that eATP operates as one key regulatory signal in the dynamic pathogen-host cross talk, with the balance of this interaction affecting the infection outcome.

## Results

### Effect of nucleotide scavengers on AP induction

To test AP induction by *S. marcescens*, EGFP-LC3-Chinese hamster ovary (CHO) cells were co-incubated with the WT strain for 120 min, after which cells were visualized by confocal microscopy. A *S. marcescens shlBA* mutant strain lacking ShlA expression was used as a negative control. AP induction by the WT strain was revealed by an EGFP-LC3 green fluorescent punctate pattern, indicative of LC3 recruitment onto nascent autophagosome membranes, as opposed to a homogenous distribution of fluorescence observed when the *shlBA* mutant strain was used. To determine whether eATP might influence the AP induction response, experiments were run in the absence or presence of an excess of enzymes known to remove eATP (35, 36).

**Figure 1:**
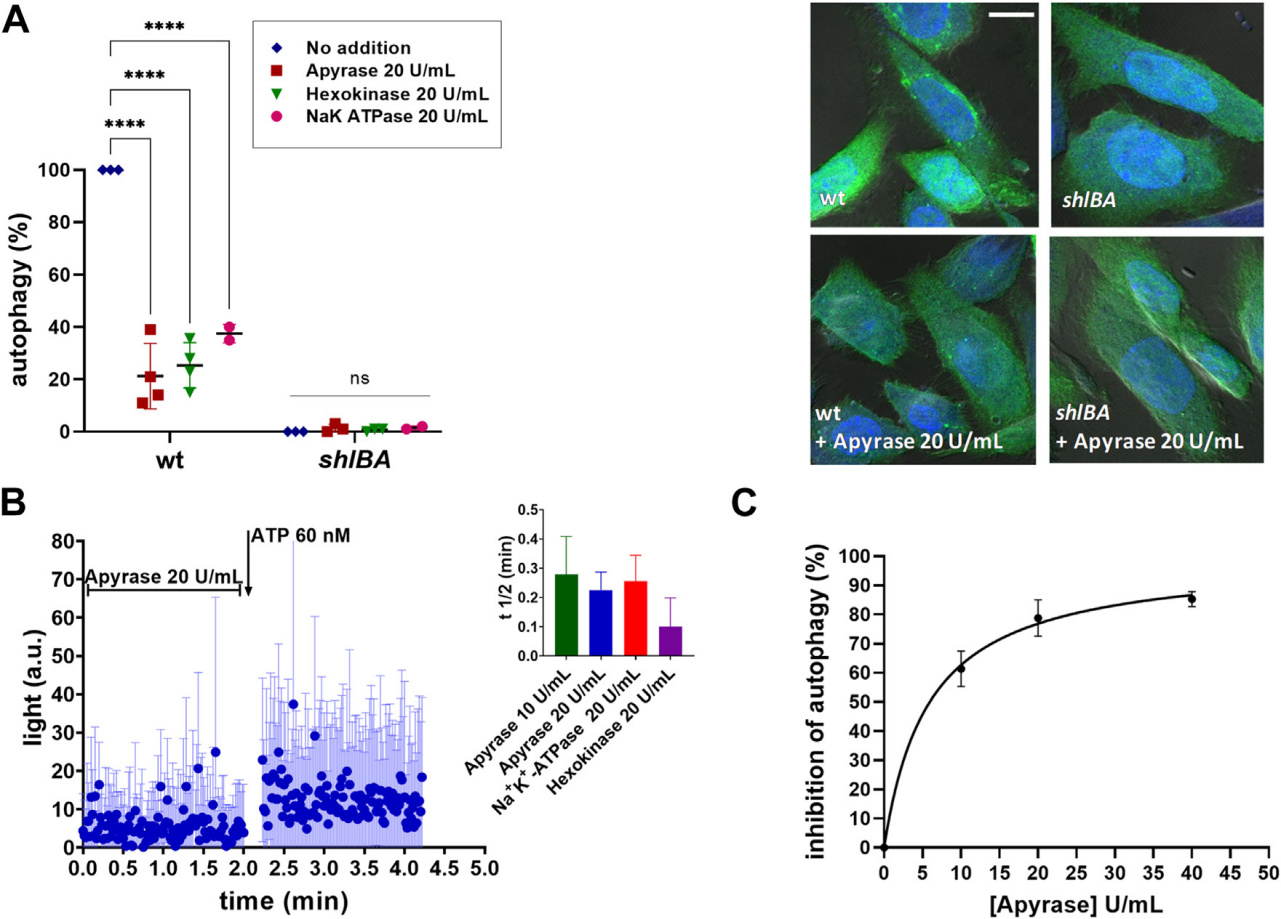
**Inhibition of ShlA-dependent AP of CHO cells caused by nucleotide scavengers.***A*, CHO-EGFP-LC3 were incubated with 20 U/ml of apyrase, hexokinase, or Na^+^,K^+^ ATPase. After 15 min, cells were coincubated with WT or *shlBA Serratia marcescens* strains. At 120 min coincubation (c.i.), cells were visualized by confocal microscopy (*right panels*) and ShlA-dependent AP was calculated relative to AP in the absence of enzymes. The scale bars represent 10 μm. *B*, eATP degradation in the presence of 20 U/ml apyrase, in the absence of cells. At 2.2 min, 60 nM ATP was added, and the eATP kinetics was quantified. [eATP] was estimated by light production a.u (arbitrary units). Similar experiments were run (Fig. S1) using hexokinase and Na^+^,K^+^-ATPase, which allowed to derive half-time (t_1/2_) values of eATP decay due to the activities of the three enzymes (inset). *C*, effect of apyrase on AP inhibition. A hyperbolic function (*continuous line*) was fitted to data. Data represent mean values ± SD of N = 4. ∗∗∗∗ denote *p* ≤ 0.0001, two-way ANOVA, and Tukey–Kramer multiple comparisons test. AP, autophagy; CHO, Chinese hamster ovary; eATP, extracellular ATP.

Enzymes were added to assay media–containing CHO cells prior to bacterial infection. Results show that apyrase, hexokinase (HK), and Na^+^,K^+^-ATPase, at 20 U/ml, were able to reduce the autophagic response by 63 to 79% (Fig. 1*A*). Experiments using HK, Na^+^,K^+^-ATPase and apyrase in the absence of cells or bacteria, but in the presence of 60 nM ATP, showed that ATP degradation is complete and rapid, with t_1/2_ amounting to 0.1 to 0.5 min (Figs. 1*B* and S1). Inhibition of AP increased nonlinearly as a function of apyrase concentration (Fig. 1*C*). A hyperbolic function was fitted to data, with K_0.5_ = 5.9 U/ml. To test the effect of nucleotides on AP in the absence of toxin, CHO cells were preincubated with 3 μM of ATP, ADP, or UTP before exposure to the *shlBA* strain. Results show that nucleotides *per se* did not induce AP.

Overall, our results imply that ATP accumulates in the extracellular medium as a consequence of CHO exposure to ShlA and this eATP triggers AP. The following experiments were designed to test this hypothesis.

### Kinetics of eATP accumulation of CHO cells exposed to *Serratia*

**Figure 2:**
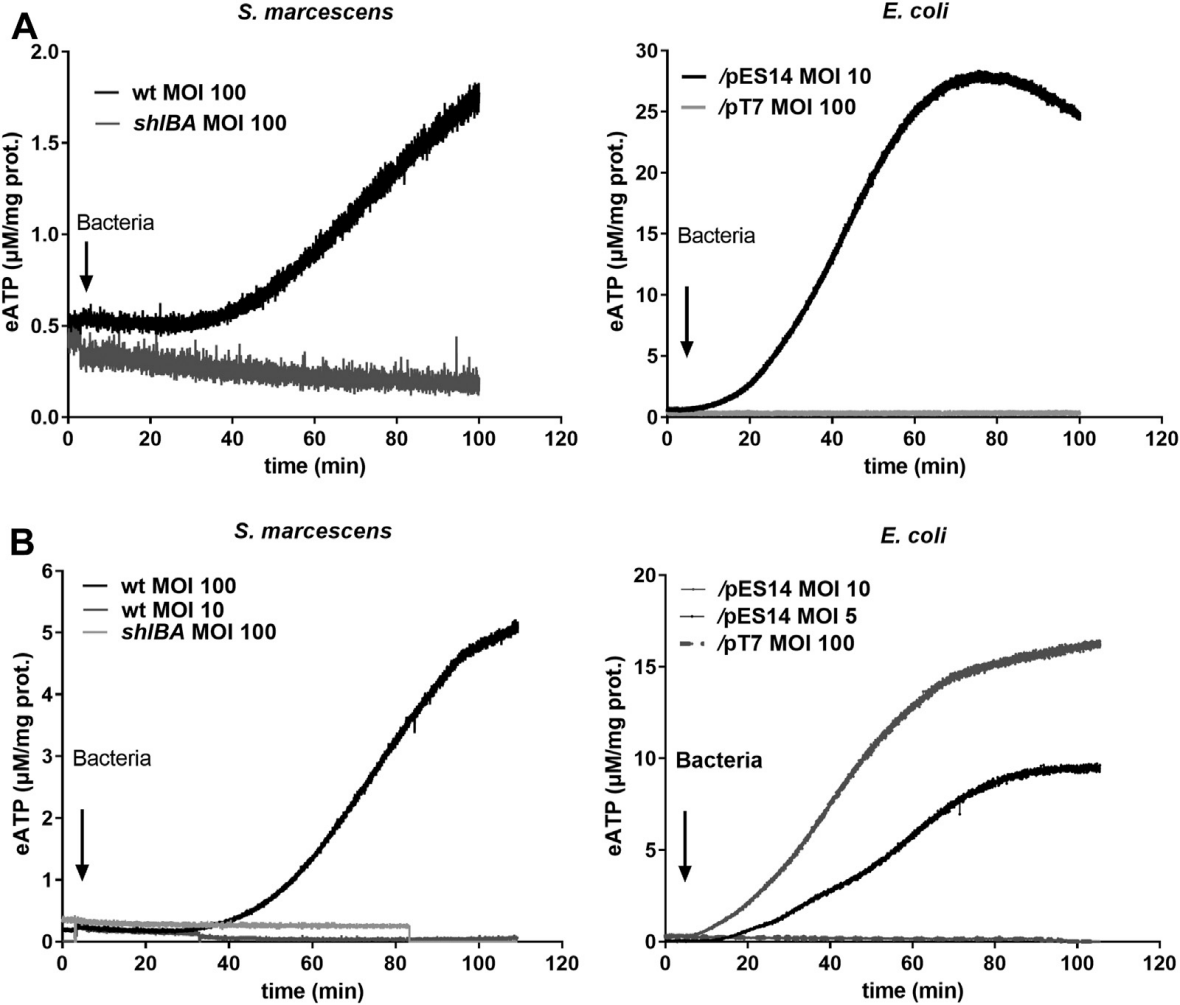
**eATP kinetics of host cells exposed to bacteria.***A*, the time course of [eATP] from CHO cells was quantified by real-time luminometry. At t = 5 min, CHO cells were exposed to *Serratia marcescens* WT or *shlBA* strains (MOI = 100) (*up*) or *Escherichia coli/*pES14 (MOI = 10) *and E. coli/*pT7 (MOI = 100) (*down*). *B*, eATP kinetics of Caco-2 cells was quantified as in (*A*). At t = 5 min, Caco-2 cells were exposed to *S. marcescens* WT (MOI = 10 and 100) (*left*) or *shlBA* mutant strains (MOI = 100) or *E. coli/*pES14 (MOI = 5 and 10) and *E. coli/*pT7 (MOI = 100) strains (*right*). In (*A*) and (*B*), [eATP] was expressed as μM/(mg. protein). Data represent mean values of N = 4 independent experiments. CHO, Chinese hamster ovary; eATP, extracellular ATP; MOI, multiplicity of infection.

Kinetics of eATP was investigated under different experimental conditions (Fig. 2). When CHO cells were exposed to WT *S. marcescens*, eATP concentration ([eATP]) remained stable for at least 30 min, and it subsequently increased nonlinearly with time, indicating that CHO cells are able to release ATP. After 100 min, [eATP] increased 3.5 ± 0.1-fold relative to basal values (Fig. 2*A*). No increase in [eATP] was detected in similar experiments using the *shlBA* strain. Although P2 receptors’ activation and extracellular ADP can modulate iATP release in other systems (37, 38), eATP kinetics of WT *Serratia*-challenged CHO cells were neither affected by 100 μM suramin (ΔATP_120min_ = 0.2 ± 0.04 *versus* control values of 0.16 ± 0.02) nor by 1 μM exogenous ADP (ΔATP_120min_ = 0.26 ± 0.06 *versus* control values of 0.16 ± 0.02).

Next, we quantified eATP kinetics of CHO cells exposed to noninvasive *Escherichia coli* W3110 strain transformed with the pES14 plasmid that harbors the *shlBA* operon. This strain displays 1.8-fold higher hemolytic activity when compared to WT *Serratia*. *E. coli* W3110/pES14 at multiplicity of infection (MOI) 10 exhibited a relatively slow [eATP] increase lag phase followed by a fast [eATP] increase to a maximum, indicating activation of ATP release. At the late phase of eATP kinetics, [eATP] decayed indicating eATP hydrolysis by ATPases. Maximum accumulation of eATP with *E. coli* W3110/pES14 at MOI 10 was 4.5-fold higher than the highest [eATP] obtained using WT *Serratia* at MOI 100. No changes in [eATP] were observed using the *E. coli* W3110 strain (Fig. 2*A*), reinforcing the notion that ShlA is the inducer of ATP efflux from CHO cells.

In the absence of host cells, bacterial [eATP] was stable, so that ShlA was not inducing bacterial ATP release (Fig. S1*F*). Exposure to melittin (a permeabilizing peptide) caused an acute ATP release from WT *Serratia*. However, we used 2 × 10^8^ bacteria for this experiment, which in the hypothetical presence of CHO cells would represent an effective MOI >2600, that is, 26-fold the MOI employed in experiments shown in Figure 2*A*. Thus, such bacterial ATP release, even under high bacteriolysis, would not affect eATP kinetics of CHO cells. This means that eATP (Fig. 2) originates entirely from iATP of CHO cells.

Cytotoxicity was assayed in CHO cells challenged by each of the four strains, *S. marcescens* WT or *shlBA* and *E. coli* W3110/pT7 (empty vector) and *E. coli* W3110/pES14. Cytotoxicity values tested by the thiazolyl blue tetrazolium bromide (MTT) colorimetric assay moderately but continuously increased (Fig. S2*A* and MTT inset). However, this increase was not related to iATP release, since it was also observed in the strains lacking ShlA expression, which did not induce changes of [eATP]. Moreover, as evaluated by a flow cytometry assay, propidium iodide uptake, a marker of cellular membrane damage, showed low values for the exposure of CHO cells to all bacterial strains, thus discarding a lytic component of ATP release (flow cytometry inset, Fig. S2*A*).

Caco-2 cells were also exposed to either WT *S. marcescens* (MOI 100) or to *E. coli* W3110/pES14 (MOI 5 or 10). Sigmoidal patterns of eATP kinetics were observed (Fig. 2*B*), indicating that Caco-2 cells release ATP when exposed to ShlA. For *E. coli* W3110/pES14, [eATP] accumulation values increased as a higher MOI was used. eATP accumulation could not be detected when either WT *Serratia* at MOI 10 or the *shlBA* strain at MOI 100 were used, probably due to the sensitivity of the methodology used to measure eATP (Fig. 2*B*).

### Mechanisms of iATP release and their relationship to AP

**Figure 3:**
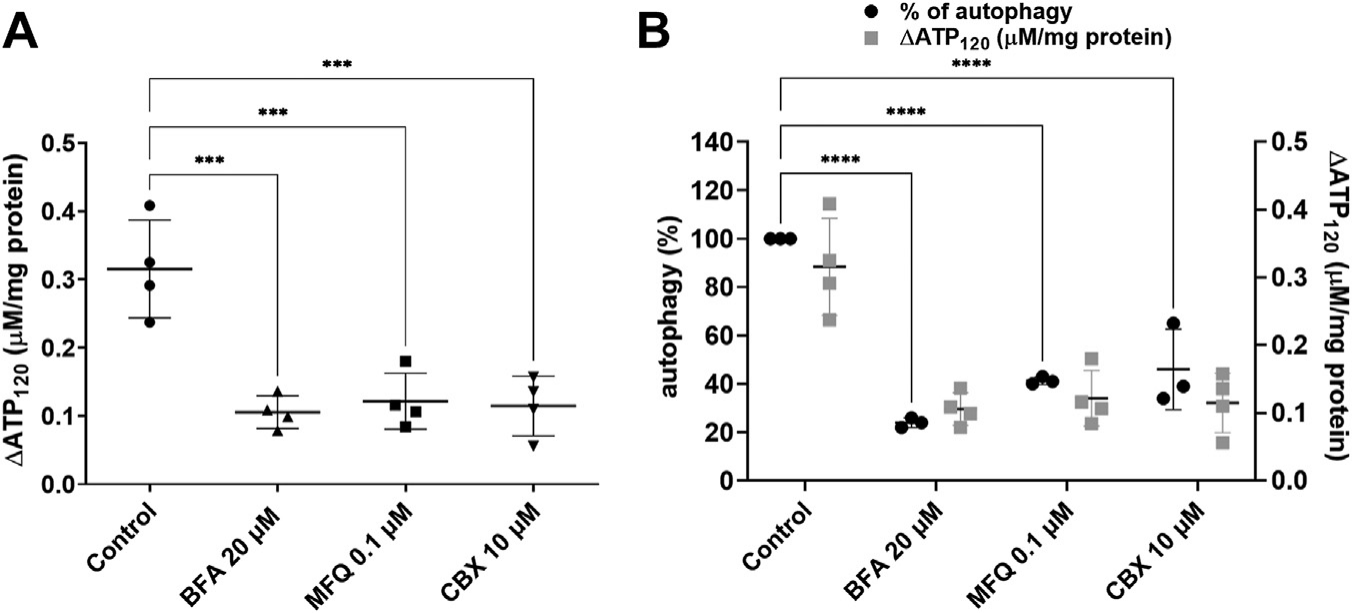
**Effect of inhibitors of iATP release on eATP and autophagy.** The time course of [eATP] from CHO cells was quantified as in Figure 2. Then (*A*) increases in [eATP] were evaluated as the difference between [eATP] at 120 min poststimulus and the basal [eATP] and are indicated as ΔATP_120_. Cells were preincubated 10 min with 10 μM carbenoxolone (CBX) or 100 nM mefloquine (MFQ), or 3 h with 0.1 μM brefeldin A (BFA). Five minutes following pretreatment, CHO cells were exposed to *Serratia marcescens* WT strain (MOI = 100). Control experiments were run in the absence of inhibitors. *B*, similar treatment as in (*A*) was applied to determine relative values of autophagy (AP, in %), quantified as described in Figure 1. Values of ΔATP_120_ taken from (*A*) are shown for a comparative purpose. CHO, Chinese hamster ovary; eATP, extracellular ATP; iATP, intracellular ATP; MOI, multiplicity of infection.

Having determined that iATP release of CHO cells is not due to cell lysis, we next treated cells with blockers of potential ATP cell membrane conduits that facilitate iATP efflux. Pretreatment with carbenoxolone (CBX) (10 μM) as well as mefloquine (MFQ) (100 nM), two well-known inhibitors of PNX1 (25, 39, 40), reduced ShlA-induced [eATP] increase by 61 to 63% (Fig. 3*A*). In addition, brefeldin A (BFA), a blocker of exocytosis, reduced [eATP] increase by 66%.

Results suggest that, in CHO cells exposed to WT *Serratia*, ATP release is mediated by PNX1 and by exocytosis.

In ShlA-challenged CHO cells, experiments using CBX, MFQ, and BFA showed parallel inhibition of AP and decrease in [eATP], with tight correlation (Fig. 3*B*).

### Hydrolysis of eATP by bacteria and CHO cells

**Figure 4:**
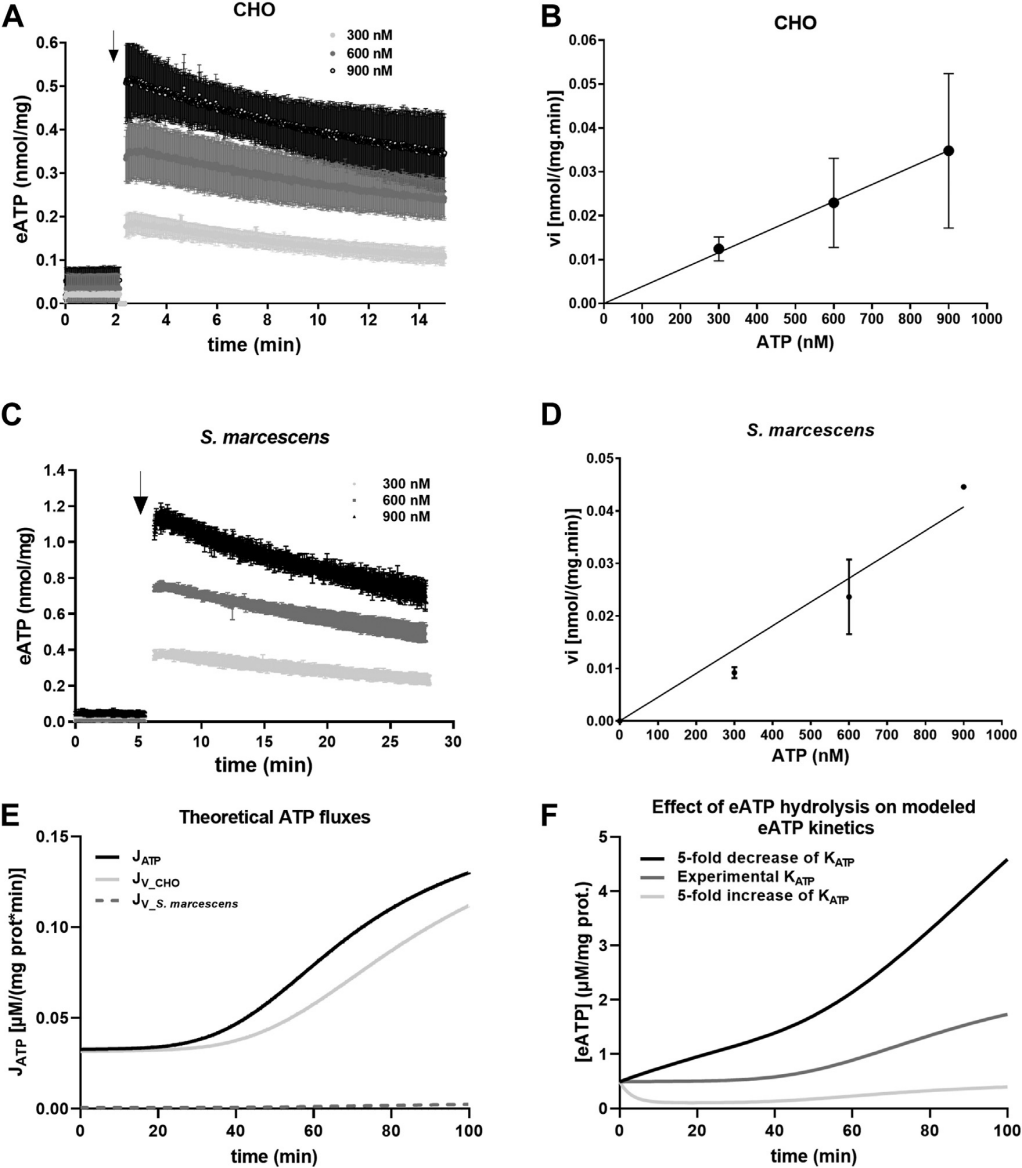
**Rates of eATP hydrolysis by CHO cells and *Serratia marcescens* and model predictions.***A* and *B*, CHO cells. *C* and *D*, WT *S. marcescens*. *A* and *C*, show eATP kinetics in the absence and presence of increasing [ATP]. *Arrows* show time of ATP addition. For (*B*) (CHO cells) and (*D*) (WT *S. marcescens*), each point ± SD of the graphs was calculated from initial velocities (vi) of eATP decay kinetics measured in (*A*) and (*C*), respectively. Linear functions were fitted to data. Data represent mean values ± SD of N = 4. *E* and *F*, results of a model showing aspects of eATP hydrolysis for CHO cells exposed to WT *S. marcescens*. Details of the model in Fig. S8. *E*, modeled fluxes of ATP. J_ATP_ = ATP efflux from CHO cells, Jv-CHO = rate of eATP hydrolysis by ectonucleotidases of CHO cells, J_v_*S.marcescens*_ = eATP hydrolysis by ATPases of *S. marcescens*. (Jv_CHO) was slightly lower than J_ATP_, and several fold higher than bacterial J_v_*S.marcescens*_. *F*, effects of changing the rate of eATP hydrolysis of CHO cells on eATP kinetics. Predictions of eATP kinetics were made considering the experimentally determined rate constant of eATP hydrolysis (“K_ATP_” set to 1), a 5-fold increase or a 5-fold decrease of K_ATP_. CHO, Chinese hamster ovary; eATP, extracellular ATP.

Kinetics of eATP shown in Figure 1*A* not only depends on iATP release (increasing [eATP]) but also on eATP hydrolysis (decreasing [eATP]). Accordingly, experiments were run to assess the capacity of bacteria and CHO cells to degrade eATP (Fig. 4). First, the kinetics of eATP was measured for CHO cells exposed to exogenous ATP. Following ATP addition at three different concentrations, acute [eATP] increases were observed, followed by decay phases (Fig. 4*A*). By fitting a monoexponential decay function to data, the initial rates of [eATP] decrease at each [ATP] could be determined. This ecto-ATPase activity (vi) increased in direct proportion to [ATP] (Fig. 4*B*). Fit of a linear function to (vi) *versus* [ATP] data provided a slope (K_ATP-CHO_), amounting to 0.77 ± 0.19 min^−1^ mg^−1^.

A similar procedure was carried out to assess eATP hydrolysis by *Serratia* (Fig. 4*C*). The addition of different concentrations of ATP to the bacterial assay medium caused an initial increase and subsequent decrease in [eATP]. After calculating initial velocities of ATPase activity at each [ATP], ATPase activity (vi) was plotted as a function of [ATP]. A linear fit to these data yielded a slope (K_ATP—*Serratia*_) of 0.91 ± 0.22 min^−1^ mg^−1^ (Fig. 4*D*).

The above results imply that, within the micromolar range where eATP accumulates in ShlA-challenged CHO cells, increases in [eATP] concentration can in principle be counteracted by eATP hydrolysis. A quantitative analysis of these results is presented in the Mathematical modeling section below.

Additional experiments were performed to test the maximal capacity of *E. coli*, *Serratia,* and CHO cells to hydrolyze nucleotides. The three systems exhibited significant hydrolysis of ATP (Fig. S3), ADP, and AMP (Fig. S3). However, neither nucleotides nor adenosine affected *Serratia* growth (Fig. S2, *B* and *C*), ruling out the potential effect of a metabolic or energetic advantage for the bacteria to induce the release of ATP from the eukaryotic cell.

### Mathematical modeling of eATP kinetics of CHO cells challenged by *Serratia*

The observed ShlA-induced eATP kinetics (Fig. 2*A*) is the result of ATP release by CHO cells and eATP hydrolysis by both ectonucleotidases of CHO cells (Fig. 4*A*) and periplasmic ATPases of *Serratia* (Fig. 4*C*). While eATP kinetics and eATP hydrolysis constitute experimental results, the rate of iATP release can be calculated using a data-driven model. In addition, once the model was fitted to experimental data, it allowed to quantify the contribution of ATP release and eATP degradation to the dynamic regulation of eATP (Fig. 4, *E* and *F*). In the model, time-dependent changes in [eATP] were expressed as:

$$\frac{\partial \left[eATP\right]}{\partial t}=J_{ATP}-J_{v-CHO}-J_{v-bact}$$

with J_ATP_ being the rate of iATP release by CHO cells, J_v-CHO_ the eATP hydrolysis by CHO cells, and J_v-bact_ the eATP hydrolysis by *Serratia.*

The predicted kinetics of the three fluxes are shown in Figure 4*E*. For CHO cells challenged by *Serratia*, J_ATP_ (*i.e.*, ATP efflux) displayed a lag phase, followed by a continuous nonlinear increase. J_ATP_ and J_v-CHO_ displayed comparable rates, while J_v-bacteria_ was much lower and therefore unable to affect eATP kinetics.

By comparing J_ATP_ with [iATP] of CHO cells (6.02 ± 1.04 mM), it was possible to calculate the energetic cost of ATP efflux in CHO cells, showing that eATP represented 1 to 2.5% of iATP (Fig. S4*A*).

The consequences of ectonucleotidase activity of CHO cells can be best observed by considering changes in K_ATP_, the kinetic constant of the substrate curve shown in Figure 4*F*. A K_ATP_ value set to 1 (1-fold of the experimental value) allows to model eATP kinetics matching the experimental results. A 5-fold reduction in K_ATP_, that is, K_ATP_ = 0.2, accelerated a sustained [eATP] increase (Fig. 4*F*), while a 5-fold increase in K_ATP_ (*i.e.*, K_ATP_ = 5) leads to very low values of eATP kinetics.

WT *Serratia* displays significant hydrolysis of eATP in the low micromolar range (Fig. 4, *C* and *D*). However, at MOI = 100, such ATPase activity has no effect on eATP kinetics of CHO cells. This is why simulating eATP kinetics at MOI = 0 or 100 provided similar results (Fig. S4*B*). For bacterial ATPase activity to affect eATP kinetics, MOI values should have to increase at least two orders of magnitude (Fig. S4*B*).

### Effects of purinergic signaling on AP induction

**Figure 5:**
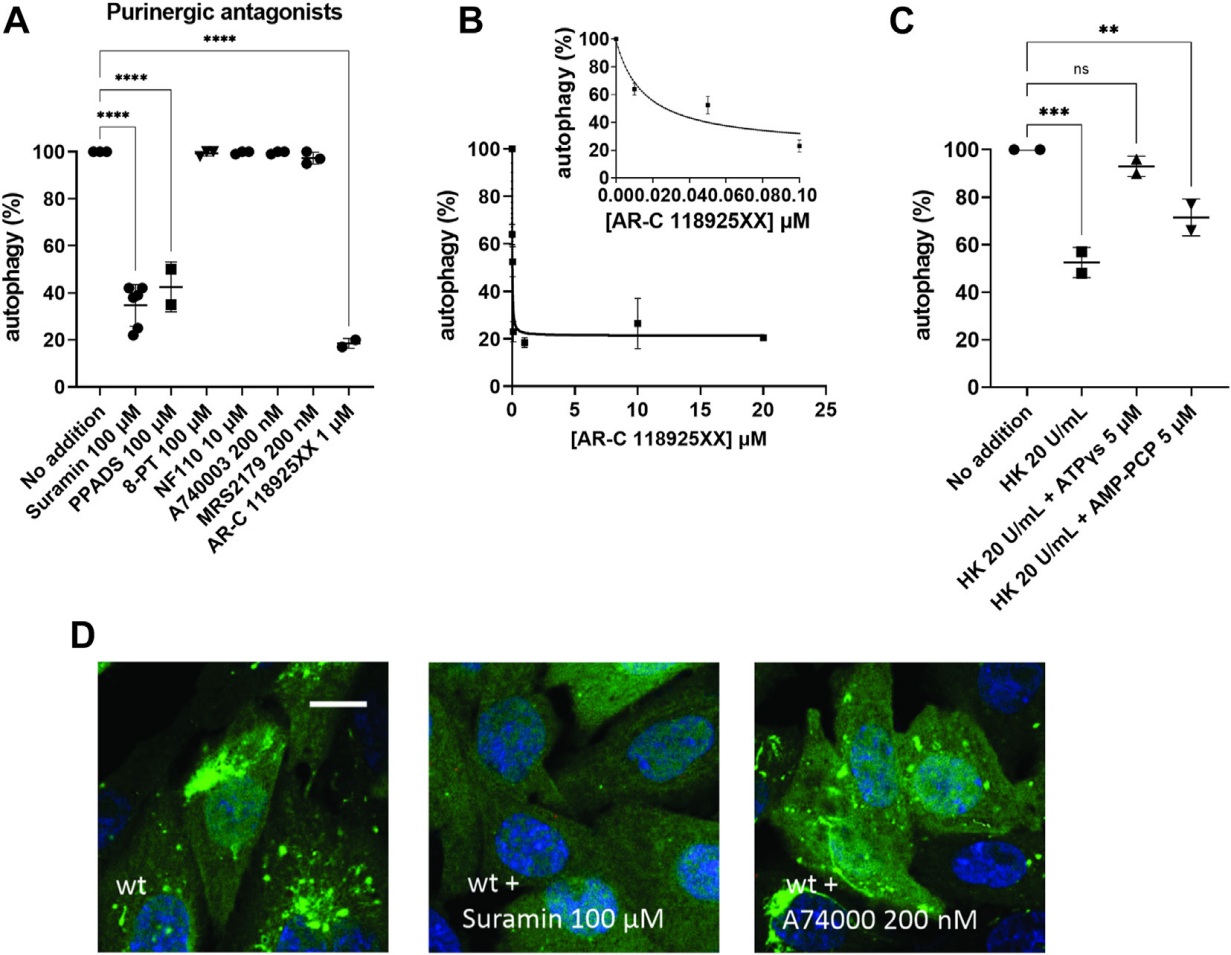
**Effect of purinergic antagonists and agonists on ShlA-dependent autophagy of CHO cells.***A*, AP (%) with suramin 100 μM, PPADS 100 μM, 8-phenyl theophylline (8-PT) 100 μM, NF110 10 μM, A740003 200 nM, 200 nM MRS2179, and 1 μM AR-C118925XX. *B*, AP (%) as a function of [AR-C118925XX]. A hyperbolic function was fitted to experimental data (0–25 μM). *C*, effects of ATP analogs on AP. CHO cells were preincubated 10 min with 20 U/ml hexokinase (HK) and then exposed simultaneously to WT *Serratia marcescens* and ATPγs or AMP-PCP. *D*, representative images corresponding to results in (*A*). CHO-EGFP-LC3 cells were incubated with suramin 100 μM or A74000 200 nM. After 15 min, cells were coincubated with WT *S. marcescens*. After 120 min c.i., cells were visualized by confocal microscopy. The scale bars represent 10 μm. Data represent mean values ± SD of N = 4 independent experiments. ∗∗∗∗ denote *p* ≤ 0.0001, ANOVA, and Tukey–Kramer multiple comparisons test. CHO, Chinese hamster ovary; c.i., coincubatino.

Because our results indicate that eATP activates AP, the blockage of one or various P2 receptors displaying high affinity for eATP is anticipated to decrease the autophagic response. Suramin or pyridoxalphosphate-6-azophenyl-2',4'-disulfonic acid, two broad spectrum P2 receptor blockers, resulted in 58% inhibition of AP (Fig. 5, *A* and *D*). Since such compounds, used at relatively high concentrations, may produce nonspecific effects on metabolic responses and signaling, a range of subtype-specific blockers were used. Blockers of neither P2X_1–3_ (NF110) nor P2X_7_ (A740003) affected AP (Fig. 5*A*). In addition, the null effect of 8-phenyl theophylline blocker (Fig. 5*A*) allowed us to discard the potential effect of adenosine (produced from eATP hydrolysis) on P1 receptors. eATP hydrolysis of CHO cells (as observed in Fig. 4) should produce extracellular ADP, which can then activate high affinity P2Y1 receptors present in various cell types (41, 42). However, the selective P2Y1 antagonist N6-methyl-2′-deoxyadenosine-3′,5′-bisphosphate (MRS2177) (43) did not affect AP induction (Fig. 5*A*). On the other hand, as low as 100 nM AR-C118925XX, a highly specific blocker of the P2Y2 receptor (44) inhibited AP by 80%. Moreover, Figure 5*B* shows that AR-C118925XX dose dependently inhibited AP, saturating potency at 1 μM. By fitting a hyperbolic function to data, AR-C118925XX inhibition displayed a K_0.5_ = 15.9 ± 5.6 nM, and a remnant AP (obtained at asymptotic maximal [AR-C118925XX]) of 21.4 ± 3.8%).

In principle, P2Y2 should be activated by low-micromolar eATP to induce AP, no matter whether the nucleotide is produced endogenously from iATP release or exogenously provided. To test this hypothesis, CHO cells were preincubated 20 min with 20 U/ml HK, a treatment capable of efficiently removing ATP (Fig. S1*C*). Under this condition, 5 μM of the slow degradable analogs ATPγs and AMP-PNP were added together with the *Serratia* challenge. Results show that, having removed endogenous eATP with HK, both nucleotide analogs increased the AP induction (*i.e.*, reverted the AP inhibition induced by HK) from 52% (HK) to 71% (HK + AMP-PCP) or to 93% (HK + ATPγs). The higher effect of ATPγs *versus* AMP-PCP agrees well with the high affinity of this analog to P2Y2 (45) (Fig. 5*C*).

Altogether, results show that in CHO cells AP induction promoted by ShlA is mediated by P2Y2 receptors. In contrast, neither suramin nor apyrase were able to reduce the canonical AP that occurs when CHO cells are challenged by starvation (Fig. S5).

**Figure 6:**
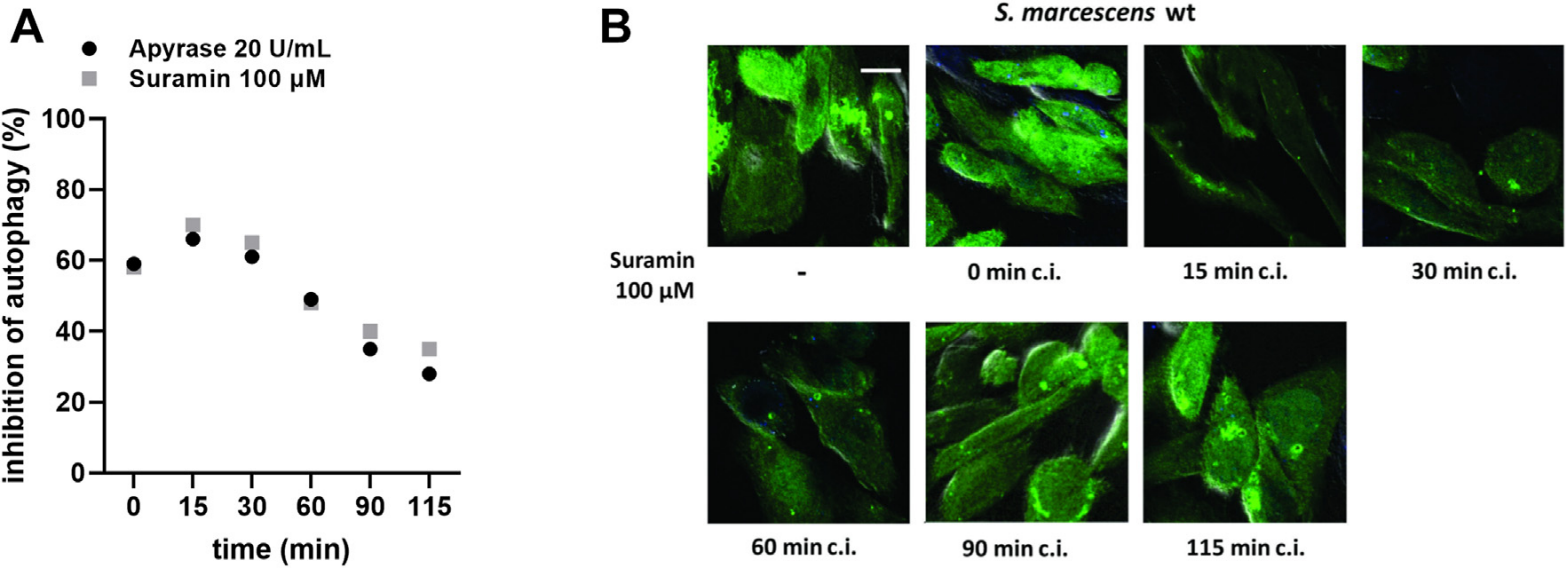
**Kinetics of autophagy inhibition in the presence of apyrase and suramin.***A*, inhibition of ShlA-dependent autophagy of CHO cells in the presence of suramin 100 μM or apyrase 20 U/ml added at different times coincubation (c.i.). CHO-EGFP-L3 cells were exposed to WT *Serratia marcescens*. At 120 min c.i., cells were fixed and visualized by confocal laser microscopy. Time-dependent inhibition of autophagy was assessed. Results are expressed as percentage inhibition relative to the autophagy caused by ShlA in the absence of treatments. *B*, representative images of CHO-EGFP-LC3 exposed to suramin. The scale bars represent 10 μm. Data represent mean values of N = 4 independent experiments. CHO, Chinese hamster ovary.

The kinetic behavior of J_ATP_ (Fig. 4*E*) is similar to the kinetics of AP inhibition provoked by addition of suramin and apyrase (Fig. 6*A*), as it can be observed when J_ATP_ and AP (in %) were plotted together *versus* time (Fig. 6*B*). Apyrase or suramin treatments are less effective as J_ATP_ increases.

### Role of integrin α5β1 on P2Y2-dependent induction of AP

Given that P2Y2 has been described to display an RGD motif that is recognized by α5β1 integrin (44), we examined whether the integrin receptor might be involved in the ShlA-dependent signaling cascade. For that purpose, before AP was induced by WT *Serratia,* CHO cells were preincubated with a peptidomimetic integrin antagonist that targets α5β1 integrin with high affinity (46), either in the absence or the presence of 20 U/ml apyrase.

**Figure 7:**
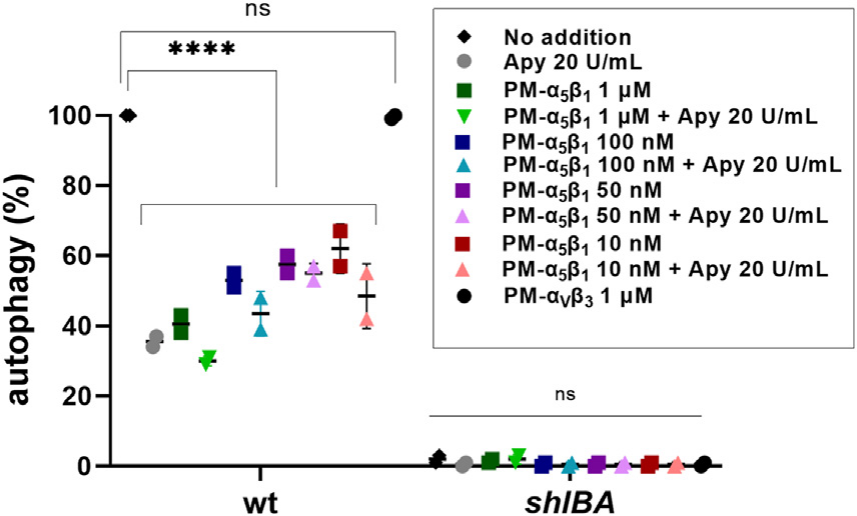
**ShlA-dependent autophagy of CHO cells in the presence of an integrin α5β1 antagonist.** CHO-EGFP-LC3 were preincubated 60 min with various concentrations of an integrin α5β1 antagonist (PM-α5β1) in the absence and presence of 20 U/ml apyrase. As a negative control, cells were preincubated in the presence of 1 μM of a αvβ3 integrin antagonist (PM-αvβ3). Following pretreatment, cells were coincubated with WT or *shlBA S. marcescens* strains. At 120 min c.i., cells were visualized by confocal microscopy. Results are expressed as the percentage of ShlA-dependent autophagy of CHO cells. CHO, Chinese hamster ovary; c.i., coincubation.

Results showed that, in the presence of WT *Serratia*, α5β1 blockage reduced AP by 60% (no apyrase) and by 70% (with apyrase) (Fig. 7). These results were similar to those obtained by P2Y receptor blockage or apyrase treatment (Figs. 1 and 5). Lower concentrations (10–100 nM) of the antagonist had reduced potency on AP inhibition (Fig. 7). No response was obtained for the different treatments when the *shlBA* strain was used (Fig. 7).

Collectively, these results indicate that the α5β1 integrin receptor is involved in the signal transduction cascade that elicits AP in CHO cells exposed to ShlA.

Integrin α5β1 may exist in a dynamic equilibrium between inactive and active states (47). We thus run experiments to verify whether α5β1 integrin was active when CHO cells were challenged by WT *Serratia*, both in the absence or the presence of eATP. For that purpose, CHO cells were exposed to 1 mM Mn^2+^, a well-known integrin activating reagent (48), both in the absence or presence of 40 U/ml apyrase. Results show that 1 mM Mn^2+^ caused a slight but not significant increase of AP (Fig. S6). This suggests that, in the presence of ShlA, α5β1 is present mostly in an active state. As observed before (Fig. 1*A*) apyrase highly reduced AP. However, addition of Mn^2+^—in the presence of apyrase—increased AP from 30 to 62%. As expected, when cells were exposed to the *shlBA* mutant strain, Mn^2+^ had no effect.

This observation suggests that eATP removal (achieved through apyrase treatment) led to the inactivation of P2Y2, thereby shifting the equilibrium of α5β1 conformations toward inactive state(s). Under this condition, Mn^2+^ causes activation, although to a smaller extent than when eATP is present (≈60% *versus* 100%).

### Induction of *Serratia* egress from the intracellular vacuole

**Figure 8:**
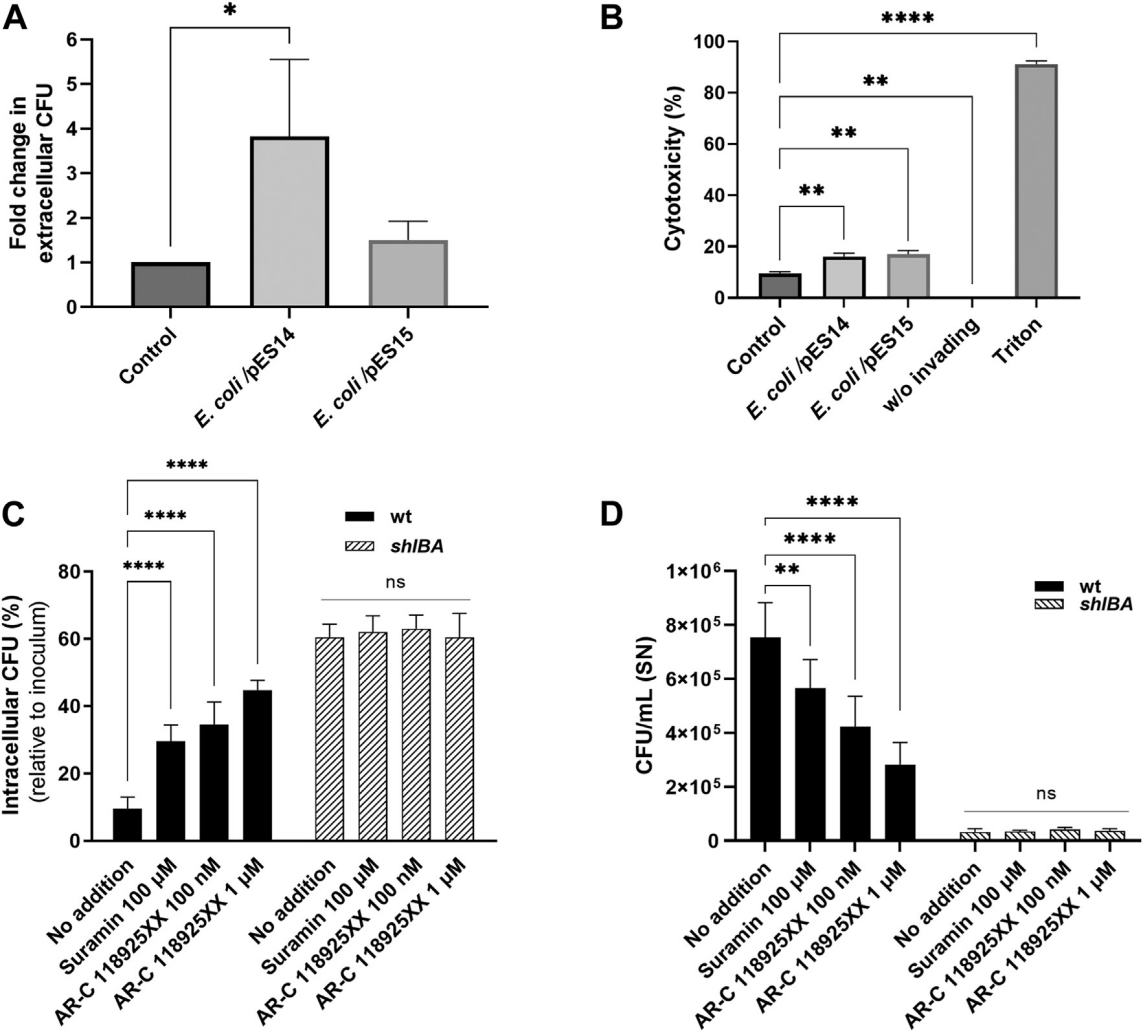
**Purinergic dependent escape of *Serratia marcescens* from CHO cells.***A* and *B*, CHO cells were infected with *S. marcescens shlBA* strain. After 60 min, extracellular bacteria were killed by gentamicin. At 240 min postincubation (p.i.), antibiotic-free medium and *Escherichia coli*/pES14 or *E. coli*/pES1*5* were added, when indicated. *A*, at 360 min p.i., CFUs of extracellularly released *shlBA* strain were determined. *B*, at 360 min p.i., MTT was added to assess cytotoxicity. Noninvaded cells treated with Triton X-100 were used as a positive control; noninvaded and untreated cells were used as a negative control. Average values ± SD of N = 3 are shown (∗*p* < 0.05; ∗∗*p* < 0.005, ∗∗∗∗*p* < 0.0001). *C* and *D*, CHO cells were infected with WT or *shlBA* strains. At 0 or 240 min p.i., suramin was added. *C*, at 360 min p.i., intracellular CFU (%) was calculated relative to the inoculum. Average ± SD of N = 3 is shown (∗*p* < 0.05). *D*, after 240 min p.i., gentamicin was eliminated by replacement of free-antibiotic medium. CFU/ml in supernatant (SN) was determined at 360 min p.i. Data represent means ± SD of N = 3. ∗∗ denote *p* ≤ 0.005 and ∗∗∗*p* < 0.001 (two-way ANOVA and Tukey–Kramer multiple comparisons test). CFU, colony-forming unit; CHO, Chinese hamster ovary.

Because we have previously demonstrated that ShlA expression was required to promote the bacterial exocytic, nonlytic egress from the invaded host cell, we examined whether this process could be linked to ShlA-dependent activation of P2Y2. We first analyzed whether the sole extracellular contact of ShlA with cells previously invaded with the *shlBA* strain could promote these intracellular mutant bacteria (otherwise unable to egress) to be exocyted. The exposure of CHO cells to a noninvading *E. coli* strain that recombinantly expresses ShlA rescued the *shlBA* strain in its escape from the invaded cell (Fig. 8*A*). We also corroborated that bacterial release was not due to a cytotoxic effect on CHO cells (Fig. 8*B*).

Next, we compared the effect of the inhibitors suramin (P2-generic) and AR-C118925XX (P2Y2-selective) on the capacity of WT *versus* the *shlBA* mutant strains to egress from CHO cells. Addition of each blocker could prevent WT strain exit, as measured by an increased percentage of intracellular bacteria (Fig. 8*C*) or by concomitant diminished bacteria released to the culture supernatant (Fig. 8*D*). Therefore, under the action of either inhibitor, the WT strain mimicked the *shlBA* mutant strain behavior, while the *shlBA* mutant inability to exit from CHO cells was not altered by the compounds (Fig. 8, *C* and *D*). We also discarded that incubation of CHO cells with 3 μM ATP, ADP, UTP, and adenosine before exposure to the *Serratia* strains affected bacterial egress (Fig. S7).

Overall, these results show that *Serratia* ShlA-dependent activation of P2Y2 receptor is an early signaling event that will promote the clearance of *Serratia* from the invaded host cell and allow bacteria to disseminate extracellularly after intracellular multiplication.

## Discussion

Results of this study show that WT *S. marcescens* induces regulated iATP release from CHO cells. The resulting eATP activates a complex signaling pathway that triggers AP and mediates a postinvasion response, provoking the nonlytic clearance of the pathogen from the host cell.

### Induction of noncanonical AP by eATP

Several lines of evidence support the role of eATP in ShlA-dependent AP. First, an excess of three unrelated exogenous enzymes capable of degrading eATP blocked the ShlA-mediated AP in host cells. Second, WT *Serratia*, but not the otherwise isogenic *shlBA* mutant strain, promoted the release of iATP from CHO cells, leading to continuous accumulation of eATP. This response was replicated by exposing cells to a noninvasive *E. coli* strain transformed with a plasmid that harbors the *shlBA* operon, which expresses ShlA and its cognate transporter ShlB (12).

Inhibition of PNX1 by low concentrations of CBX (whose binding site is clearly identified in the channel (49), and MFQ, as well as blockage of exocytosis by BFA, strongly suppressed the ShlA-dependent AP response. Because WT *Serratia* did not induce lysis on CHO cells, these results indicate that ATP release occurs by regulated processes.

In addition to the response on CHO cells, we also observed that ShlA significantly enhanced ATP release from Caco-2 cells, a model of enterocytes and colonic adenocarcinoma (50). This is particularly important considering that *S. marcescens* is an opportunistic pathogen with an ample range of human host cells, including intestinal epithelial cells (51).

### Energetics of eATP regulation

eATP signaling did not impose an energy burden to CHO cells, since eATP accumulation required approximately 2% of total iATP. However, results using the *E. coli* strain that overexpresses ShlA, and thus displays higher cytotoxic activity than WT *Serratia*, show that the energy cost for host cells can be higher, potentially contributing to energy depletion of the host. Previous reports showed that ShlA exposure to different eukaryotic cells led to vacuolation due to irreversible ATP depletion, sometimes leading to cytolysis (52). In this study, we show that subtler noncytolytic changes mediated by purinergic signaling are sufficient to elicit the autophagic response.

A low energy requirement by the host to produce eATP is in line with the low [eATP] required to activate ATP-sensitive P2Y receptors (53). Moreover, it also agrees well with the infection cycle of *Serratia* in which invasion and intracellular proliferation is followed by bacterial egress without compromising the viability of the host cell (20).

### P2Y2 signaling

eATP may signal onto one or various P2 receptors of the host cell. By using broad spectrum and subtype-specific P2-blockers, we showed that AP in CHO cells was highly blocked by AR-C11895, a highly selective antagonist for P2Y2 (54) that displays a potency in the midnanomolar range (54). This agrees well with an app. K_0.5_ (about 16 nM, see Fig. 5*B*) for AP blockage in CHO cells. Moreover, ShlA-dependent eATP accumulation in CHO cells is congruent with both, the reported affinity for P2Y2, and the above reported low energy requirement of CHO cells. In addition, our results using suramin and AR-C118925 show that, in addition to AP, ShlA-promoted bacterial non-lytic egress also depends on purinergic signaling. Furthermore, we verified a tight coupling between the temporal pattern of AP inhibition and either the suramin or apyrase blockage of P2Y2-dependent ATP release.

### Downregulation of eATP-P2Y2 signaling by eATP hydrolysis

Model-dependent fit to experimental results showed that, following exposure of CHO cells to *Serratia*, [eATP] kinetics depended on both iATP release from host cells, and [eATP] hydrolysis by ecto-ATPase activity of CHO cells. Under the analyzed experimental conditions, and in contrast to other bacteria that are able to hydrolyze substantial eATP even at low MOI (55), rates of eATP hydrolysis by *Serratia* ATPases were very low and thus unable to alter ShlA-dependent eATP kinetics of CHO cells.

### Role of α5β1 integrin

Like other Gαq-coupled P2Y receptors, activation of P2Y2 stimulates the canonical Gαq/phospholipase C/inositol triphosphate signaling axis, leading to release of calcium from intracellular stores. This route appears functional in CHO cells, since micromolar ATP and UTP are able to increase inositol triphosphate and mediate calcium oscillations (56, 57). However, the link between Gαq activation and AP in different cell systems is not clear. Although downstream signaling through PI3K/Akt/mTor was shown to inhibit the LC3-I to LC3-II conversion (58), thereby inhibiting the AP response, in CHO cells, we showed that wortmannin, a blocker of PI3K, does not interfere with noncanonical AP induction of WT *Serratia* (21).

Another signaling route activated by P2Y2 involves the interaction of Arg-Gly-Asp motif within its first extracellular loop with αVβ3/5 and α5β1 integrins, two members of the RGD-recognizing family (44). Because CHO cells exhibit a functional α5β1 (but no β3 integrins), we used a peptidomimetic compound shown to specifically antagonize α5β1 integrin (46). This antagonist dose dependently decreased ShlA-dependent AP up to 40%, implying that P2Y2, activated by eATP, may transactivate α5β1 integrin, leading to AP induction. Alternatively, given that P2Y2 is able to activate five distinct signaling pathways (44), an outside-in signaling modulating α5β1 integrin cannot be discarded.

Because BFA interferes with the central vacuolar system traffic, it may not only alter eATP accumulation and subsequent P2Y2 activation but also the transit of integrins to and from the cell membrane (59), thus affecting transactivation. We have previously shown that intracellular WT *Serratia* promotes a ShlA-dependent calcium mobilization that leads to dynamic modulation of the cytoskeleton required to induce the exocytosis of bacteria (22). This would be consistent with ShlA promoting P2Y2 transactivation of integrins that results in the Gα12- and Gαo-dependent activation of Rho, Rac, and Cdc42, which give rise to cytoskeletal rearrangements (44).

### Reversible injuries to the plasma membrane

It has been shown that injuries to the plasma membrane provoked by bacterial toxins can induce an AP-related process that involves macropinocytosis. This mechanism promotes the maintenance of the plasma membrane integrity and encompasses removal of damaged material and repair of the membrane structure (60). The ShlA-dependent AP induction is reversible and unrelated to the biogenesis of the autophagic-like *Serratia*-containing vacuoles (21). Therefore, it is tempting to speculate that ShlA-induced P2Y2 activation is required to trigger an AP-related healing process as a short-term response. In fact, we herein show that the ShlA-mediated AP induction does not overlap with the canonical starvation-induced pathway. In principle, brief and reversible injuries occurring in CHO cells exposed to ShlA should lead to pulsed increases of [eATP] (complementing MFQ- and BFA-sensitive ATP efflux), which may contribute to the observed residual, MFQ/BFA-tolerant, component of ATP release.

**Figure 9:**
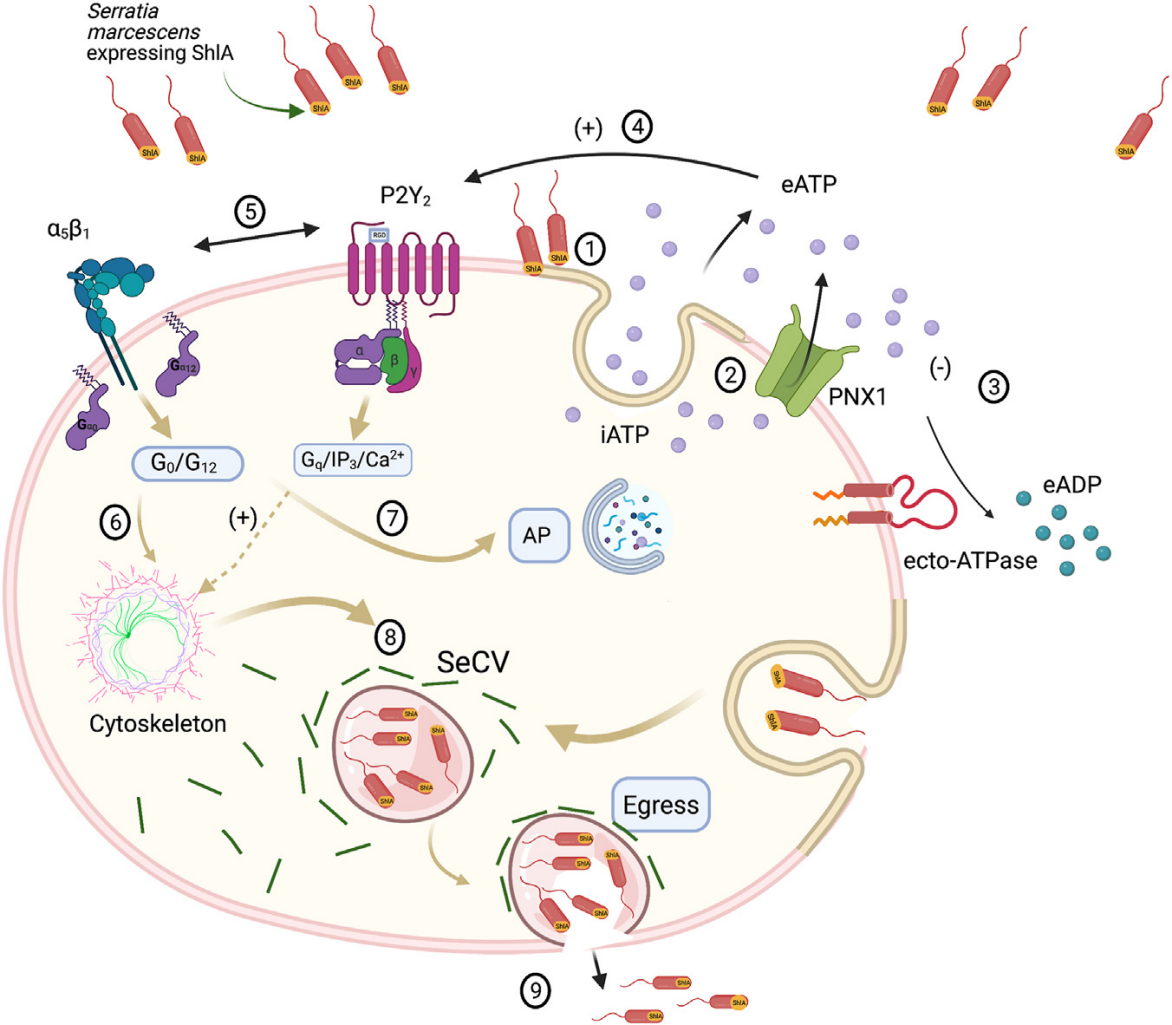
**Graphical model explaining the effects of eATP-P2Y2-α5β1 signaling on autophagy and *Serratia marcescens* escape.** CHO cells challenged by *Serratia* (that expresses *ShlA*) release iATP by exocytosis (1), and pannexin 1 (PNX1) (2). Alternatively, exocytosis and PNX1 may be involved in a single ATP-release mechanism. The resulting accumulation of eATP has at least two fates: (i) eATP can be concentration dependently hydrolyzed by ecto-ATPase activity of the host cell, producing extracellular ADP (eADP) (3) and (ii) eATP can activate with high-affinity PY2 receptors functionally present in CHO cells (4). In turn, activated P2Y2, *via* its Arg-Gly-Asp motifs in the first extracellular loop, is able to transactivate α5β1 integrin (5). Alternatively (not visualized), outside-in signaling of P2Y2 might indirectly modulate α5β1 integrin. On speculative basis, the P2Y2–α5β1 interaction is necessary for P2Y2 to activate Go/G12 signaling routes, thus inducing two distinct phenotypes: an early AP response (7) and postinvasion cytoskeletal rearrangements allowing *Serratia,* replicating in an intracellular vacuole (SecV) (8), to be exocyted (9). A potential endocytotic entry of *Serratia* into CHO cells is illustrated. CHO, Chinese hamster ovary; eATP, extracellular ATP; iATP, intracellular ATP.

To summarize our main findings a graphical model of our postulated eATP-P2Y2-integrin signaling cascade mechanism modulating ShlA-dependent phenotypes is provided in Figure 9. At least two different transport systems facilitate ShlA-dependent iATP release from CHO cells. eATP can then be partially hydrolyzed by ecto-ATPase activity of CHO cells and activate P2Y2 receptors. Next, P2Y2–α5β1 interaction would activate Go/G12 promoting AP and also trigger a nonlytic egress and dissemination of intravacuolar bacteria from the host cell.

### Final remarks

Considering that nosocomial infections due to *S. marcescens* are hard to treat, our results suggests that strategies aimed at interfering with purinergic signaling would be able to suppress the ability of the host cell innate immune response to counter the effects of ShlA and hinder the intracellular traffic of *Serratia* that leads to egress and spread within the host. Numerous compounds targeting purinergic receptors and/or integrins in clinical contexts are available to treat atherosclerosis, excessive inflammation, cancer, retinal neovascularization, and in age-related macular degeneration (61, 62, 63), so that repurposing these agents or a rational development of new inhibitor molecules can be foreseen as promising therapeutic alternatives to treat *Serratia* infections.

## Experimental procedures

Bacterial strains and plasmids are listed in Table S1.

### Materials

Alpha-Minimal Essential Medium (α-MEM), Dulbecco's modified Eagle's medium F12, and Earle's Balanced Salt Solution cell culture media were obtained from Invitrogen. Fetal calf serum was purchased from Internegocios S.A. Kanamycin (50 μg/ml), chloramphenicol (20 μg/ml), and ampicillin (100 μg/ml); MTT, firefly luciferase, suramin, PPADS, 8-phenyl theophylline, NF110, HK, apyrase, ATP, ADP, AMP, UTP, and suramin were purchased from Sigma-Aldrich. LIVE/DEAD BacLight Bacterial Viability kit and 4',6-diamidino-2-phenylindole were purchased from Molecular Probes.

### Bacterial and cell culture

Bacteria were grown in LB medium supplemented with antibiotics, overnight, at 30 °C. CHO cell line (CHO-K1, obtained from ATCC) or the derived stably transfected CHO cells overexpressing EGFP-LC3 (EGFP-LC3-CHO) were grown in α-MEM supplemented with 10% fetal calf serum at 37 °C and 5% CO_2_. Caco-2 cells (ATCC) were grown in Dulbecco's modified Eagle's medium F12 (Gibco) containing 4.5 g/l glucose (Sigma-Aldrich) supplemented with 10% v/v fetal bovine serum, 2 mM L-glutamine (Sigma-Aldrich), 100 U/ml penicillin, 100 μg/ml streptomycin, and 0.25 μg/ml fungizone (Invitrogen) in a humidified atmosphere of 5% CO_2_ at 37 °C. When indicated, overnight LB medium cultures of WT *S. marcescens* were diluted 1/100, washed and inoculated in LB medium, α-MEM or M9 medium supplemented with ATP, UTP, or adenosine. *A*_600nm_ readings were determined using an Epoch 2 microplate spectrophotometer (BioTek). The means and SDs for triplicate analysis were calculated.

### AP assay

The AP assay was performed as described (20). EGFP-LC3-CHO cells were cultured in 24-well plates until they reached 50% confluence. *S. marcescens* or *E. coli* cultures were washed once with PBS, and an appropriate volume was added to each well to reach a MOI of 2. Plates were centrifuged for 10 min at 1000 rpm and incubated for 2 h at 37 °C and 5% CO_2_. Then, the cells were washed five times with PBS and fixed with 0.5 ml 3% paraformaldehyde in PBS. When indicated, before exposure to bacteria, CHO cells were pretreated with various blockers of purinergic receptors, or with the nucleotide-scavenger enzymes apyrase, HK, and Na^+^,K^+^-ATPase.

To quantify AP, images of cells were acquired *in vivo* by confocal fluorescence microscopy, using a Zeiss LSM880 confocal microscope (immersion oil objective 63x, na 1.4) coupled to Zen Black 2.1 sp3 software (https://www.zeiss.com/microscopy/en/products/software/zeiss-zen.html). Postacquisition image analysis was performed using the ImageJ software (NIH; https://imagej.nih.gov/ij/download.html). At least 200 cells were analyzed for each condition. The results for each experiment are the average of an assay performed in triplicate and independently repeated three times.

### Gentamicin protection assay (egress assay)

The gentamicin protection assay was performed as described (21). All infection assays were done at MOI = 10 for CHO cells. Percentage of intracellular colony-forming unit (CFU) was calculated relative to the inoculum. To quantify bacteria in the extracellular medium of invaded cells, gentamicin-containing medium was replaced by free antibiotic medium (22). At indicated time points, the supernatant was recovered and serially diluted. CFUs in supernatant were determined on LB agar plates, and CFU/ml was calculated. The results for each experiment are the average of an assay performed in triplicate and independently repeated at least three times.

Cell viability was determined by the MTT reduction assay and propidium iodide uptake as described before (20). Hemolytic activity assays were performed as previously described (20).

### eATP and iATP measurements

ATP was measured by real-time luminometry as described before (64). Measurements of eATP were carried out with CHO and Caco-2 cells alone, or coincubated with bacteria. Aliquots containing 75,000 cells (with or without bacteria) were incubated in 100 μl of PBS medium. Results were expressed as [eATP] at every time point of a kinetic curve (*i.e.*, eATP kinetics), with [eATP] expressed as μM/(mg protein).

Increases in [eATP] were evaluated as the difference between [eATP] at a fixed time point post stimulus and the basal [eATP] and are indicated as ΔATP_120_ (*i.e.*, 120 min post stimulus).

The (iATP) content of CHO cells and *S. marcescens* was estimated in real-time measurements as previously described (33). ATP values were expressed as iATP concentration.

### Hydrolysis of extracellular nucleotides

Maximal hydrolysis rates of ATP, ADP, and AMP were determined by the malachite green method as described (65). Cells (30,000–60,000/300 μl) were exposed to 500 μM of ATP, ADP, and AMP. The content of Pi was determined at different times. For bacteria, 100 μl aliquots of the bacterial suspension (10^9^/ml) were withdrawn as duplicate at different times. After fitting an exponential function to data, initial velocities were calculated and expressed as app Vmax in nmol Pi/μg min. The rate of eATP hydrolysis of CHO cells and *S. marcescens* at low-ATP concentrations was determined by real-time luminometry (33). ATPase activity was expressed as (vi) in nmol/mg min.

### Data analysis

Statistical analysis was performed using one-way ANOVA or two-way ANOVA and Tukey-Kramer Multiple Comparisons test or *t* test as appropriate with an overall significance = 0.05. Asterisks in the plots denote the values among the treatment groups in which a statistically significant difference was determined.

For experimental results of Figure 4*B* (AP *versus* [AR-C118925XX]), a hyperbolic function of the form: $y=\frac{a.b}{b+x}+yo$ was fitted to data, with "y" being AP (%), “yo” the asymptotic maximal value of AP inhibition, and "b" (K_0.5_) representing the concentration of AR-C118925XX at which a semimaximal inhibition of AP is obtained.

Details of the mathematical model used to quantify the dynamics of eATP regulation are given in Figure 6*D*.

## Data availability

All data are contained within the manuscript.

## Supporting information

This article contains supporting information (12, 20, 22, 66, 67).

## Conflict of interest

The authors declare that they have no conflicts of interest with the contents of this article.
