# Supplementary material for: ShlA toxin of Serratia induces P2Y2- and α5β1-dependent autophagy and bacterial clearance from host cells
Source: J Biol Chem. 2023 Jul 30;299(9):105119. doi: 10.1016/j.jbc.2023.105119 (PMC10474472; doi:10.1016/j.jbc.2023.105119)
Supplement: Supporting Information [file mmc1.docx]

**
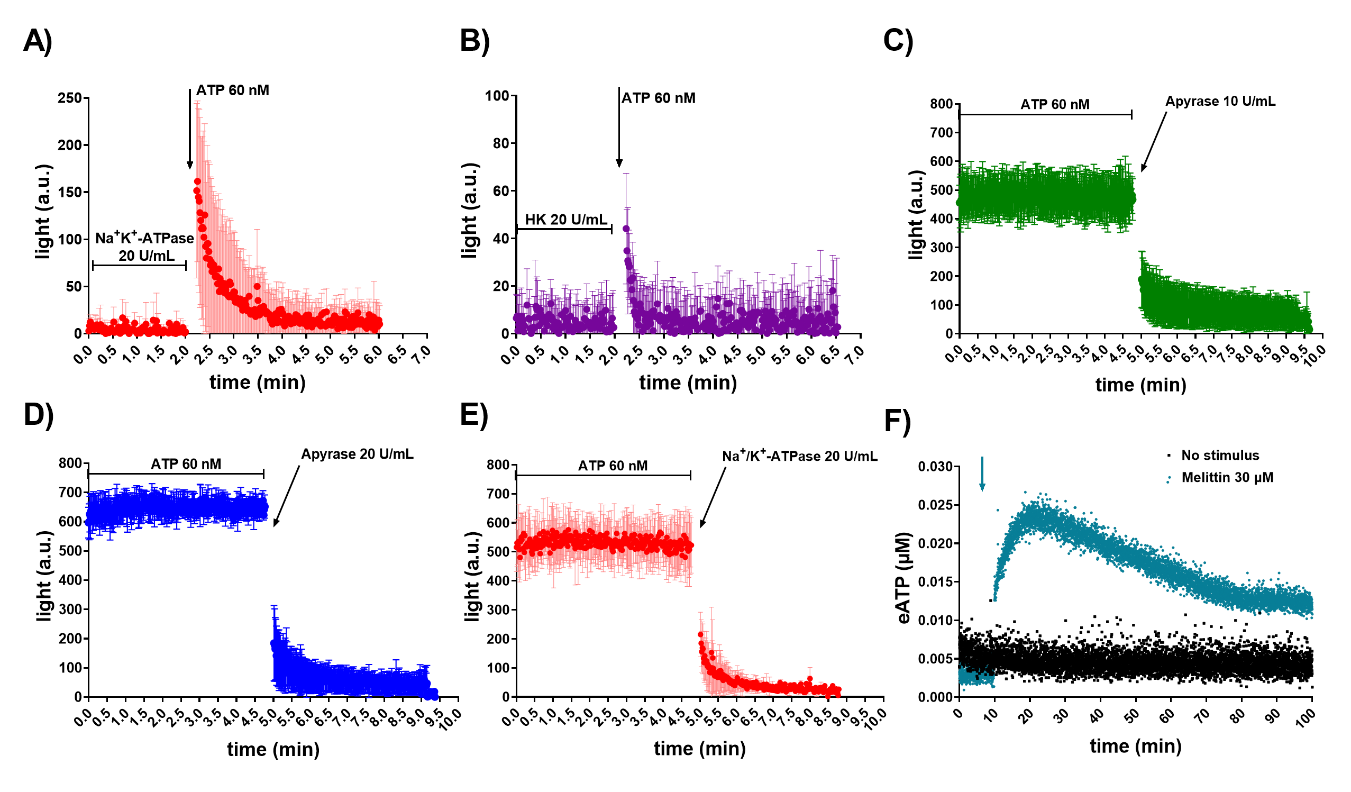
**

**Fig. S1. Effect of exogenous enzymes on eATP degradation.** Changes in [eATP] were estimated by the emission of light produced by the luciferase-luciferin system (real-time luminometry), in the absence of cells. **A-B)** Assay medium contained, 20 U/mL Na,K-ATPase (A) or 20 U/mL Hexokinase (B). At 2.2 min, 60 nM ATP was added, and the kinetics was followed for another 2 min. **C-E)** At 0 min, 60 nM ATP was added. 5 min later, 10 or 20 U/ml apyrase (C-D) or 20 U/mL Na,K-ATPase (E) were added, and ATP kinetics was followed for another 4 min. Data represent mean values ± SD of *N* = 4 independent experiments. **F)** eATP kinetics of wild type *Serratia*. The time course of [eATP] from wild-type *Serratia* was quantified by real-time luminometry. Experiments were performed in the absence of stimulus (black symbols), or in the presence of 30 μM melittin (turquoise symbols). The arrow indicates the addition of melittin. Contents of eATP were expressed as μM for 2x10^8^ bacteria suspended in 100 μl of phosphate-buffered saline (DPBS) medium. Data represent values of *N* = 3 independent experiments.

**
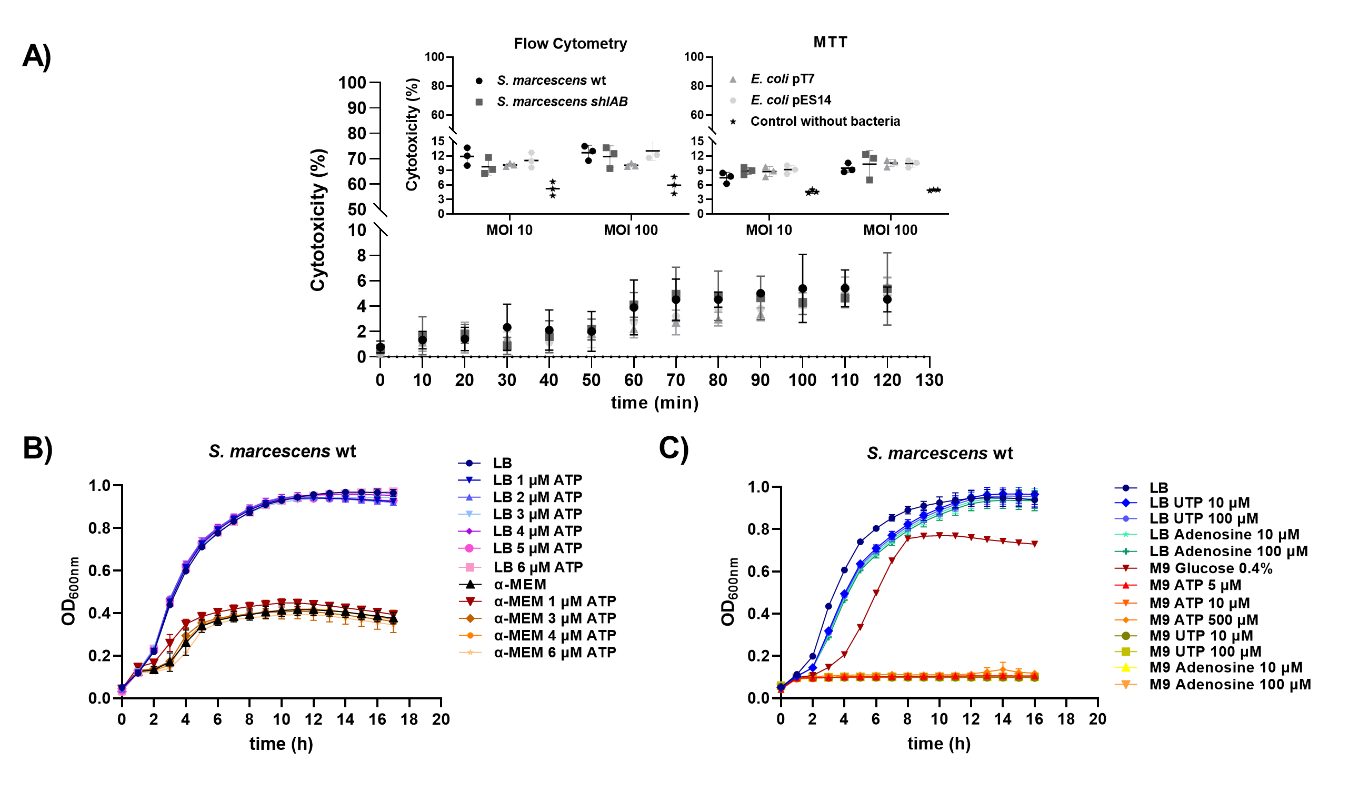
Fig. S2. CHO cell viability and growth curves of wild-type *S. marcescens***. **A)** Cytotoxicity of CHO cells. CHO cells were co-incubated with *S. marcescens* or *E. coli* strains under identical conditions as in A). Cytotoxicity was estimated by the MTT method. Cells treated with Triton X‐100 were included as the positive control and non-invaded cells were used as negative control. The average ± SD of *N* = 3 Independent experiments is shown. Inset: Cytotoxicity assay estimated by propidium iodide (PI) uptake (analyzed by Flow cytometry) and the MTT method. EGFP-LC3-CHO- cells were infected with *S. marcescens* WT or *shlBA* mutant strains*, E. coli*/pT7 or *E. coli*/pES14 strains at 25 ºC in DPBS. Measurements were made at 240 min co-incubation (c.i.). Cells treated with Triton X‐100 were included as the positive control and non-invaded cells were used as the negative control. Percentages of each population were calculated relative to the total number of cells. At least 30,000 cells were analyzed for each sample. The average ± SD of *N* = 3 independent experiments is shown. *denotes *p* ≤ 0.05, ** *p* ≤ 0.005, and *** *p* < 0.001 (two-way ANOVA and Tukey‐Kramer Multiple Comparisons test)**. B and C)** Bacteria were grown in LB medium, α-MEM medium or M9 minimal medium, in the absence or presence of glucose, adenosine and/or different nucleotides. Growth curves **(B and C)** are plotted as OD600nm values vs time (h). Experiments were run at 37 ºC under 200 rpm agitation. The average of *N* = 3 independent experiments is shown.

**
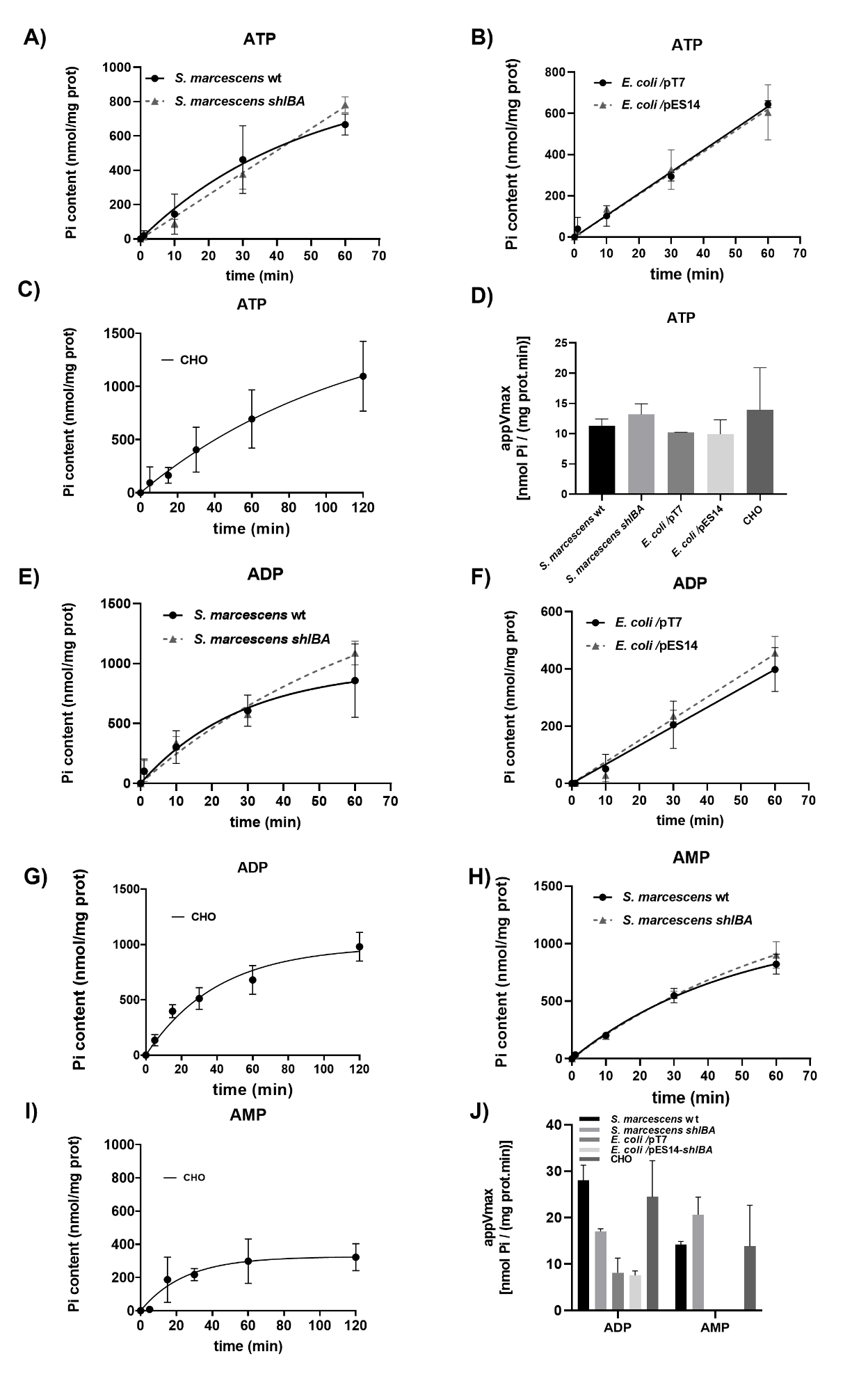
Fig. S3. Rates of ATP, ADP and AMP hydrolysis by *S. marcescens*, *E. coli* or CHO cells.** Cells and bacteria were exposed to 500 µM of ATP, ADP or AMP, and the time course of extracellular Pi accumulation was quantified. Pi production was measured using **(A, E and H)** WT and *shlBA S. marcescens* strains exposed to ATP, ADP or AMP respectively. **(B and F)** *E. coli*/pT7 and *E. coli*/pES14 strains exposed to ATP or ADP respectively. **(C, G and I)** CHO cells exposed to ATP, ADP or AMP respectively. The lines represent the fitting of monoexponential functions to experimental data. Results are expressed as Pi (nmol/mg protein) and are means ± SD with N = 4 independent experiments run in duplicate. **(D)** Values of apparent maximal Ecto-ATPase activity (appVmax) were derived from initial rates of monoexponential functions fitted to data of **(A-C).** **(J)** Values of apparent maximal Ecto-ADPase and Ecto-AMPase activities (appVmax) were derived from initial rates of monoexponential functions fitted to data of **(E-I)**. Results are expressed as [nmol Pi/(mg protein. min)] and are means + SD of N = 4 independent experiments.

**
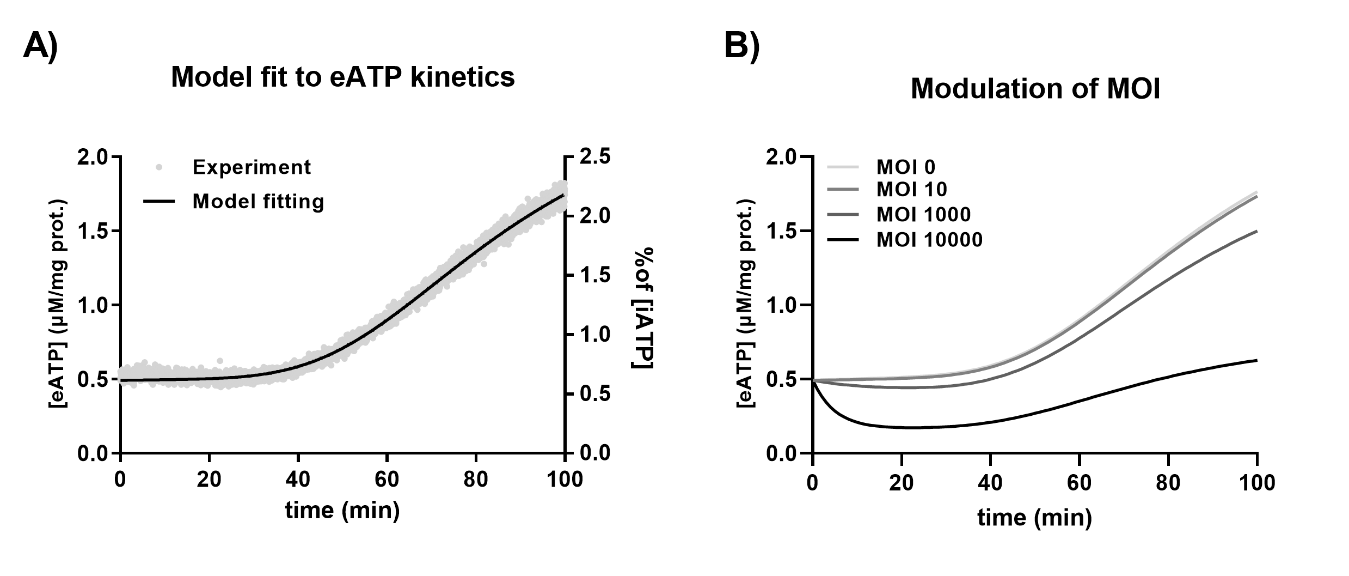
**

**Fig. S4. Modeling of eATP kinetics.** Theoretical results of a mathematical model showing various aspects of eATP regulation for CHO cells exposed to wild-type *S. marcescens*. **A)** Model fit to experimental data (gray symbols) is represented by a continuous line. Extracellular ATP (eATP) is expressed as concentration (left ordinate), or as percentage of intracellular ATP (right ordinate). **B)** Effects of bacterial concentration on eATP kinetics of CHO cells. Predictions of eATP kinetics were made considering increasing MOI values from 0 to 10,000.

Details of the model:

The following equation was fitted to eATP kinetics:

$\left[ eATP \right]=\frac{c{(\frac{x}{a})}^{5}+b(\frac{x}{a})}{e({\frac{x}{a})}^{5}+d{(\frac{x}{a})}^{3}+1}+f$ Eq. 1

where a, b, c, d, e and f are the parameters of the equation that were optimized to achieve a good fit to experimental results. Optimal values of the parameters were a=474.7 min, b=2.05 x 10^-4^ µM/µg, c= 22 µM/µg, d=246.9, e=9645 and f=4.928 x 10^-3^ µM/µg.

To calculate $\frac{\partial\left[ eATP \right]}{\partial t}$, Eq. 1 was derived as a function of x. $\frac{\partial\left[ eATP \right]}{\partial t}$ is equal to the balance of eATP generating reaction (iATP release rate, represented by J_ATP_) and eATP consuming reactions (eATP hydrolysis by CHO cells and by *Serratia* represented by J_v-CHO_ and J_v-bact_, respectively). The value of J_v-CHO_ at any time was calculated from the experimental data of [eATP] vs time as follows:

$J_{v-CHO}=K_{ATP-CHO}[eATP]$ Eq. 2

where the value of K_ATP-CHO_ is 0.064 $\frac{\mu M eATP hydrolized}{\mu g prot \mu M eATP min}$ and was measured in Fig. 4B. The value of J_v-bact_ at any time was calculated from the experimental data of [eATP] vs time as:

$J_{v-bact}=K_{ATP-bact}[eATP]$ Eq. 3

where the value of K_ATP-CHO_ is 0.0013 $\frac{\mu M eATP hydrolized}{\mu g prot \mu M eATP min}$ and was measured in Fig. 4D. The value of J_ATP_ at any time was calculated as follows:

$J_{ATP}=\frac{\partial[eATP]}{\partial t}-(J_{v-CHO}+J_{v-bact})$ Eq. 5


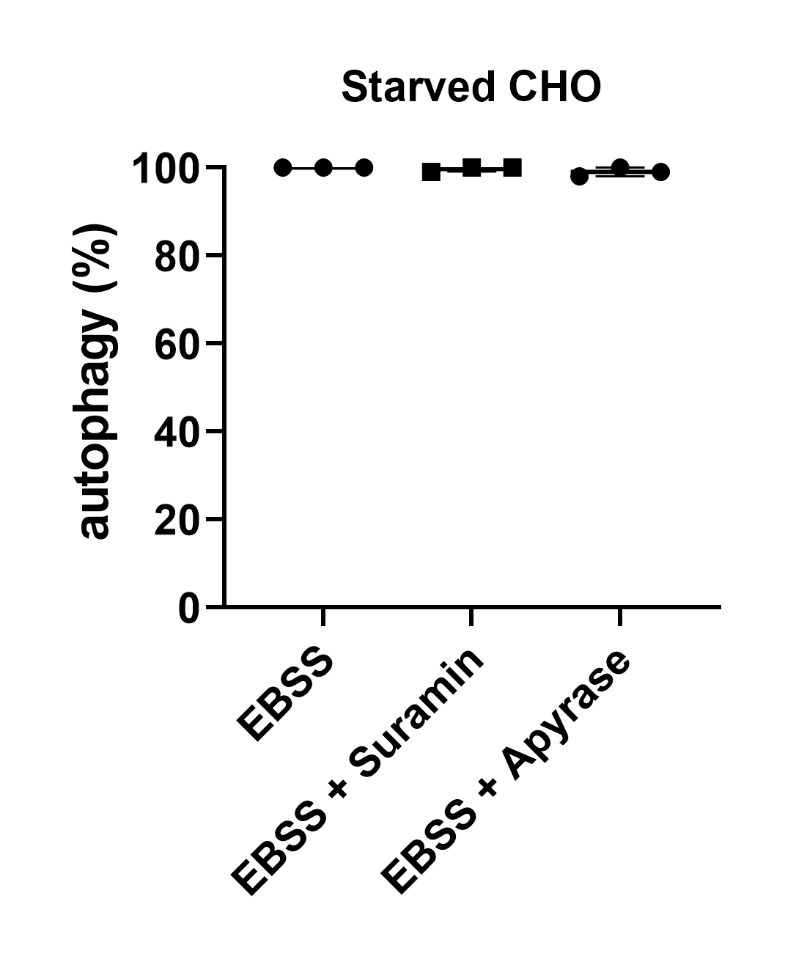


**Fig. S5. Effect of suramin or apyrase on autophagy caused by starvation in CHO cells.** Autophagy (%) of CHO cells induced by starvation in the absence of presence of either 100 μM suramin or 20 U/ml apyrase. Values are expressed as percentage of autophagy levels obtained in the presence of the minimal culture medium EBSS, used to induce starvation in CHO cells. Data represent mean values ± SD of *N* = 3 independent experiments. **** denote *p* ≤ 0.0001, ANOVA and Tukey‐Kramer Multiple Comparisons test.


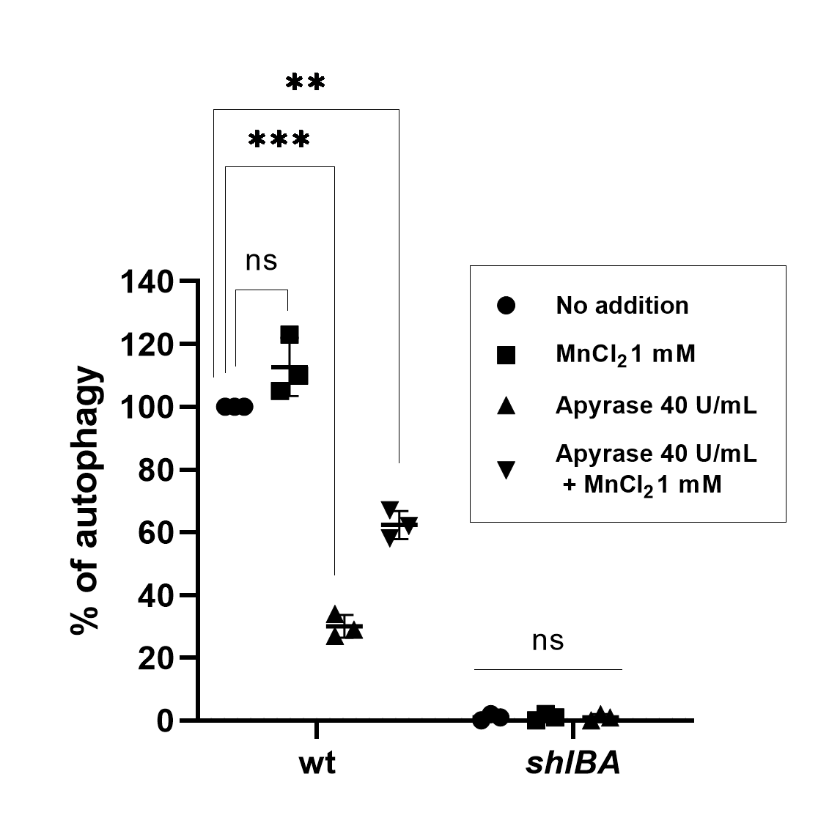


**Fig. S6.** **Autophagy of CHO cells in the presence of Mn^2+^**. CHO-EGFP-LC3 were incubated 10 min with 1 mM MnCl_2_ in the absence and presence of 40 U/mL apyrase. Subsequently, cells were co-incubated with *S. marcescens* WT or *shlBA* strains. The Apyrase + MnCl_2_ treatment was not tested on the *shlBA* strain. At 120 min c.i, cells were visualized by confocal microscopy. Results are expressed as the percentage of ShlA-dependent autophagy of CHO cells. Data represent mean values ± SD of *N* = 3 independent experiments. *denotes *p* ≤ 0.05, (two-way ANOVA and Tukey‐Kramer Multiple Comparisons test)**.**

**
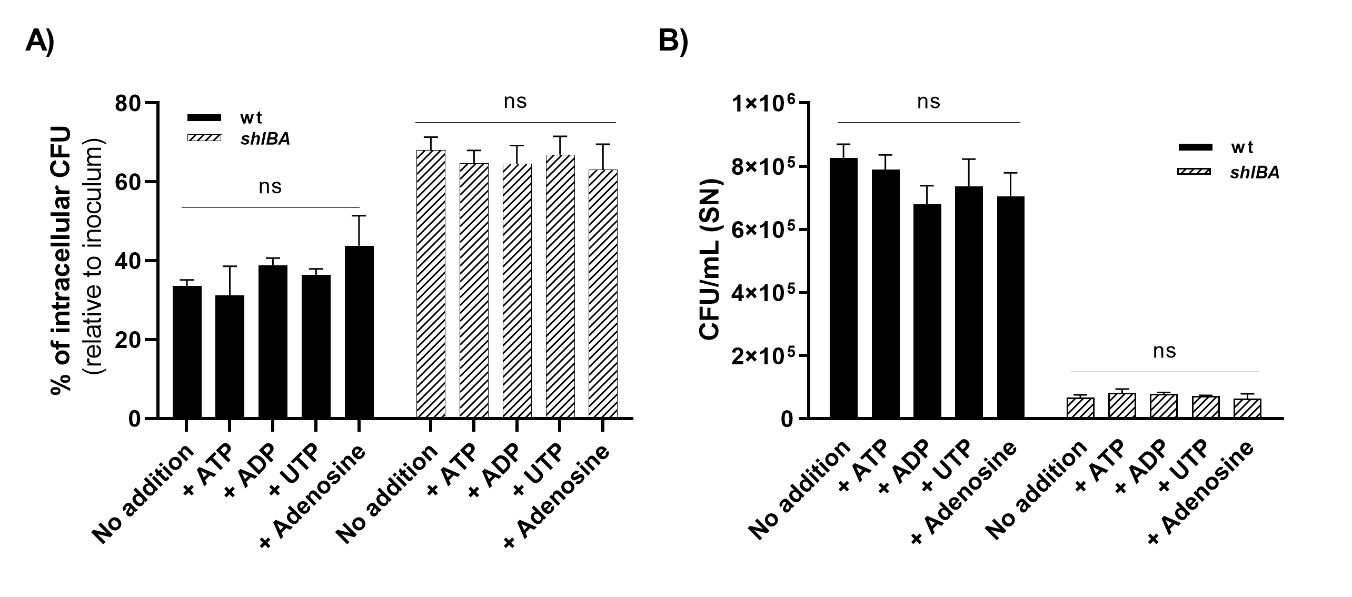
**

**Fig. S7. Effect of nucleotides and adenosine on bacterial egress**. CHO cells were infected with *S. marcescens* WT or *shlBA* mutant strain at MOI = 10. At t=0 min post-incubation (p.i.) 3µM of ATP, ADP, UTP or adenosine was added. **A)** At 360 min p.i., intracellular CFU (representing intracellular replication) was determined. The percentage of intracellular CFU was calculated relative to the inoculum. The average ± SD of *N* = 3 independent experiments is shown (* *p* < 0.05). **B)** After 240 min p.i., gentamicin was eliminated and replaced by a free‐antibiotic medium. CFU in supernatants per mL (representing bacterial egress) was determined at 360 min p.i., respectively. Data represent mean values ± SD of *N* = 3 independent experiments. *denotes *p* ≤ 0.05, (two-way ANOVA and Tukey‐Kramer Multiple Comparisons test)**.** SN: supernatant.

**Table S1. Bacterial strains and plasmids used in this study.**

| **Strains** | **Genotype and/or comments** | **Source or reference** |
| --- | --- | --- |
| ***S. marcescens*** |  |  |
| wild-type | *S. marcescens* RM66262; clinical isolate | (1) |
| *shlBA* | *shlBA*::Kan^R^ | (2) |
| wild-type/pmCherry | *S. marcescens* RM66262/  pBBR1MCS::mCherry | (2) |
| *shlBA*/pmCherry | *shlBA*::Kan^R^/pmCherry | (2) |
| ***E. coli*** |  |  |
| W3110/pT7 | T7-5 empty vector; Amp^R^ | (3) |
| W3110/pES14 | pT7–5::*shlBA*; Amp^R^ | (3) |
| W3110/pES15 | pT7–5::*shlA*; Amp^R^ | (3) |
| **Plasmids** | | |
| pmCherry | pBBR1MCS::mCherry | (2) |
| pT7 | T7-5 empty vector; Amp^R^ | (4) |
| pES14 | pT7-5:*shlBA*; Amp^R^ | (5) |
| pES15 | pT7-5:*shlA*; Amp^R^ | (5) |

**References**

1. Bruna, R. E. R. E., Revale, S., Véscovi, E. G. E. G., Mariscotti, J. F. J. F., García Véscovi, E., Mariscotti, J. F. J. F., Garcia Vescovi, E., and Mariscotti, J. F. J. F. (2015) Draft Whole-Genome Sequence of Serratia marcescens Strain RM66262, Isolated from a Patient with a Urinary Tract Infection. *Genome Announc.* **3**, e01423-15

2. Di Venanzio, G., Lazzaro, M., Morales, E. S., Krapf, D., and García Véscovi, E. (2017) A pore-forming toxin enables Serratia a nonlytic egress from host cells. *Cell. Microbiol.* **19**, 1–12

3. Di Venanzio, G., Stepanenko, T. M., and García Véscovi, E. (2014) Serratia marcescens ShlA pore-forming toxin is responsible for early induction of autophagy in host cells and is transcriptionally regulated by RcsB. *Infect. Immun.* **82**, 3542–3554

4. Tabor, S., and Richardson, C. C. (1985) A bacteriophage T7 RNA polymerase/promoter system for controlled exclusive expression of specific genes. *Proc. Natl. Acad. Sci.* **82**, 1074–1078

5. Poole, K., Schiebel, E., and Braun, V. (1988) Molecular characterization of the hemolysin determinant of Serratia marcescens. *J. Bacteriol.* **170**, 3177–3188
